# Nontargeted and targeted metabolic profile of metabolic syndrome patients: a study based on Yi and Han populations in Yunnan

**DOI:** 10.3389/fendo.2025.1488099

**Published:** 2025-05-14

**Authors:** Yanmei Ji, Ni Guo, Wenjun Li, Xianyu He, Mengyao Dao, Ni Meng, Dan Zhou, Haitao Tian, Ting Pi, Xiaofeng Zong, Qing Xiong, Zhongjuan Wang, Xingfang Jin

**Affiliations:** ^1^ Department of Cardiovascular Surgery, Yan’an Hospital Affiliated to Kunming Medical University, Clinical Medical Research Center for Cardiovascular Disease of Yunnan Province, Kunming, Yunnan, China; ^2^ Department of Pharmacy, Yan’an Hospital Affiliated to Kunming Medical University, Kunming, Yunnan, China; ^3^ Department of Endocrinology, Yan’an Hospital Affiliated to Kunming Medical University, Kunming, Yunnan, China

**Keywords:** metabolic syndrome, metabolomics, amino acids, Yi population, minority

## Abstract

**Objective:**

Ultra-high-performance liquid chromatography-time-of-flight mass spectrometry (UHPLC-TOF-MS) was employed to analyze serum metabolites and metabolic pathways associated with metabolic syndrome (MS) in the Yi and Han populations of Yunnan.

**Methods:**

Participants included individuals diagnosed with MS and healthy controls from the Yi and Han populations of Yunnan. Serum nontargeted and amino acid-targeted metabolomics analyses were conducted to identify differential serum metabolites (DEMs) and metabolic pathways associated with MS pathogenesis in these two ethnic groups.

**Results:**

Nontargeted metabolomics analysis revealed 2,762 DEMs in the MS group of the Han population, while 1,535 DEMs were identified in the MS group of the Yi population [variable importance in projection (VIP)>1, *P*<0.05]. Venn analysis highlighted common and unique DEMs between the two populations. KEGG pathway analysis identified seven significantly enriched pathways in the Han group and five in the Yi group, primarily involving amino acid synthesis and metabolism. To investigate the role of amino acids in MS, serum levels of 71 endogenous amino acids were quantified. In the MS group of the Han population, 19 differential amino acids were identified, significantly enriched in pathways related to D-glutamine and D-glutamate metabolism, as well as cysteine and methionine metabolism. In the Yi population, six differential amino acids were identified, with significant enrichment in D-glutamine and D-glutamate metabolism, sulfur metabolism, and valine, leucine, and isoleucine biosynthesis.

**Conclusion:**

Our study investigates metabolic differences in metabolic syndrome (MS) between Yi and Han populations through nontargeted and targeted metabolomics approaches, identifying both common and unique metabolites and metabolic pathways associated with MS, especially amino acid metabolic disorders, including glycine, serine, and threonine metabolism, D-glutamine and D-glutamate metabolism, which may play critical roles in regulating different metabolic dysfunctions and worth further exploration in MS pathogenesis, which might provide insights for the effective prevention and treatment of MS in various populations.

## 1 Introduction

Metabolic syndrome (MS) is a cluster of clinical conditions defined by central obesity, insulin resistance, hypertension, dyslipidemia, and elevated glucose levels (1). With global population aging and significant changes in lifestyle and diet, the prevalence and burden of MS are rising at an alarming rate (2–4). As a constellation of cardiovascular risk factors, MS impacts multiple organ systems, substantially increasing the risk of type 2 diabetes (T2D), coronary heart disease, stroke, and other disabilities, thereby posing a major public health challenge (1, 5, 6). Despite its clinical significance, the pathophysiological mechanisms of MS remain complex and poorly understood (6–9). Current research focuses on concentric obesity, lipotoxicity, and insulin resistance (10). However, it remains contentious whether distinct metabolic disorders arise from separate pathologies or represent facets of a broader, interconnected pathogenic process (11).

Metabolomic profiling, which systematically analyzes the metabolome, offers valuable insights into biological responses to endogenous and exogenous stimuli, with great potential for identifying biomarkers and elucidating disease mechanisms (12–14). Numerous metabolites have been linked to obesity (15), hypertension (15), T2D (16), dyslipidemia (17), and MS (18–20). Studies consistently report abnormalities in serum metabolites and metabolic pathways in individuals with MS, particularly those involving serum amino acid dysregulation (21, 22). For instance, a study in American and Japanese populations identified 18 shared metabolites associated with MS, primarily amino acids involved in branched-chain amino acid metabolism, glutathione synthesis, aromatic amino acid metabolism, gluconeogenesis, and the tricarboxylic acid (TCA) cycle (19). Similarly, research on the Han population revealed significantly higher plasma concentrations of isoleucine, leucine, valine, tyrosine, tryptophan, and phenylalanine in individuals with MS compared to those without (11). Dysregulated amino acid metabolism appears to be a critical feature in the onset and progression of MS across different ethnic groups.

Variations in dietary patterns, lifestyles, and genetic backgrounds among ethnic groups may contribute to disparities in the prevalence and metabolic characteristics of MS (8, 18, 20). However, the underlying mechanisms remain incompletely understood. Cross-cultural and multiethnic studies could provide valuable insights into the biochemical processes central to MS etiology. Yunnan province, notable for its ethnic diversity, includes the Yi population, which numbers approximately 5.07 million and represents the largest minority group in the region (http://stats.yn.gov.cn/Pages_22_3951.aspx). Yi individuals, as one of the oldest ethnic groups, predominantly adhere to a traditional lifestyle characterized by consistent dietary habits, intra-ethnic marriage, and genetic homogeneity. These factors make them particularly suited for investigating the interplay of genetic, dietary, and environmental influences on the prevalence of metabolic diseases (9, 10). Variations in dietary patterns, geographical environments, genetic backgrounds, and lifestyles (23–25) contribute to differing prevalence rates of MS, overweight and obesity, hypertension, and hypertriglyceridemia between Yi and Han populations (8). These differences suggest potential disparities in plasma metabolite profiles, which may underlie the distinct pathogenesis of MS in these two groups. However, no studies to date have specifically examined the metabolite signatures associated with MS in the Yi and Han populations.

In this study, non-targeted metabolomics was utilized to identify the serum metabolic signatures of MS in two ethnic groups from Yunnan. To complement this, targeted amino acid metabolomics was performed, enabling a comprehensive analysis of serum metabolite profiles associated with MS across these ethnically distinct groups. The findings aim to advance understanding of the unique metabolic characteristics underlying MS in different populations.

## 2 Materials and methods

### 2.1 Participants and sample collection

This study’s subjects were initially enrolled in a cross-sectional investigation of chronic diseases conducted by our research group in Yunnan from 2019 to 2023. The cohort included 1,250 participants: 905 Yi individuals, 307 Han individuals, and 39 from other ethnic groups (26, 27). Data on socio-demographics, smoking and drinking history, chronic medical conditions, and medication use were collected during the survey. Trained personnel performed anthropometric measurements and physical examinations, including blood pressure assessments. Blood samples were collected after a 12-hour fasting period and centrifuged at 3,500 rpm for 15 minutes. Portions of the serum samples were used for blood lipid analysis and liver and kidney function assessments, while the remaining aliquots were stored at –81°C. The study adhered to national regulations, institutional policies, and the Helsinki Declaration, receiving approval from the Research Ethics Committee of Yan’an Hospital affiliated to Kunming Medical University (approval no. 2023-060-01). All participants provided informed consent.

### 2.2 Study design

#### 2.2.1 Inclusion and exclusion criteria

**Figure 1:**
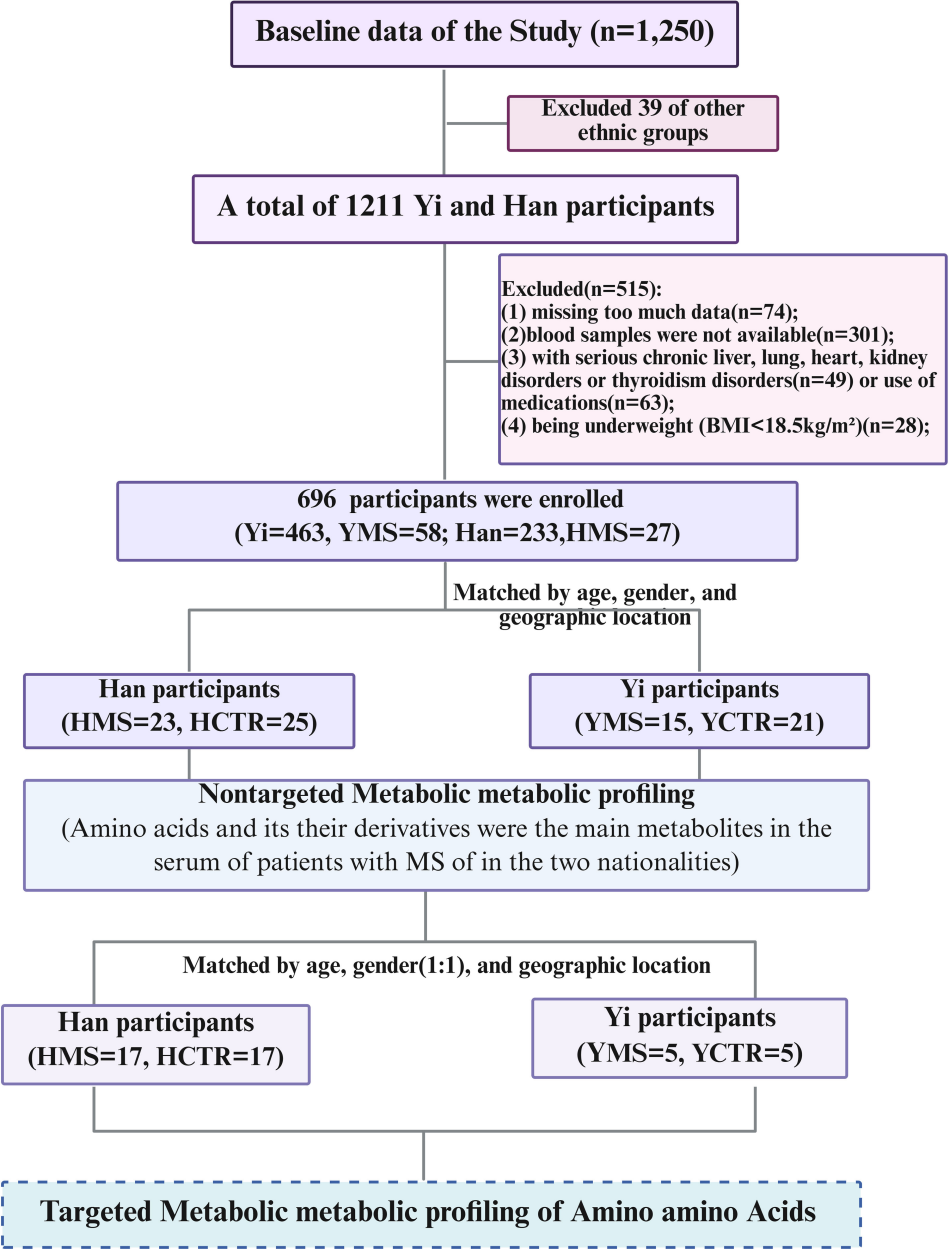
Flowchart of the study design. Figure was created with BioRender.

Eighty-four subjects were selected for untargeted metabolomics analysis, including 38 individuals with MS (15 Yi [YMS] and 23 Han [HMS]) and 46 healthy controls (21 Yi [YCTR] and 25 Han [HCTR]). Inclusion criteria required participants to (1): be aged ≥18 years, (2) reside in Yunnan Province for over five years, (3) provide informed consent, and (4) present with three or more metabolic disorders as defined by the 2004 criteria of the Chinese Diabetes Society (28). These disorders included a body mass index (BMI) ≥25.0 kg/m² (overweight/obesity), systolic blood pressure (SBP) ≥140 mmHg or diastolic blood pressure (DBP) ≥90 mmHg, fasting plasma glucose (FPG) ≥6.1 mmol/L, triglycerides (TG) ≥1.7 mmol/L, or high-density lipoprotein cholesterol (HDL-C) levels <1.0 mmol/L in females or <0.9 mmol/L in males (dyslipidemia). Healthy controls exhibited no metabolic disorders. Exclusion criteria encompassed prior use of medications for hyperglycemia, dyslipidemia, or hypertension, being underweight (BMI<18.5kg/m²), as well as the presence of cancer, advanced liver disease (Child–Pugh classes B/C), significant lung conditions (chronic obstructive pulmonary disease, chronic bronchitis, emphysema, asthma, or pneumonia), severe heart disease (New York Heart Association classes II–IV), renal impairment (eGFR <60 mL/min) (29–31), hyperthyroidism or Hypothyroidism), missing too much data or blood samples were not available. MS patients were matched with healthy controls by age (± 3 years), gender, and geographic location, a detailed case selection process is illustrated in **Figure 1**.

#### 2.2.2 Chemicals and reagents

Methanol (LC-MS, CNW Technologies), acetonitrile (LC-MS, SIGMA-ALDRICH), ammonium acetate (LC-MS, SIGMA-ALDRICH), ammonium hydroxide (LC-MS, CNW Technologies), double-distilled water (ddH_2_O, Watsons), acetic acid (LC-MS, SIGMA-ALDRICH), 2-propanol (LC-MS, CNW Technologies), acetone (LC-MS, CNW Technologies), sodium bicarbonate (NaHCO_3_, LC-MS, CNW Technologies), hydrochloric acid (HCl, LC-MS, CNW Technologies), and standard samples were used. The instruments included a Vanquish UHPLC system (Thermo Fisher Scientific, MA, USA), a Thermo Altis TSQ Plus mass spectrometer (Thermo Fisher Scientific, MA, USA), a Heraeus Fresco17 centrifuge (Thermo Fisher Scientific), an analytical balance (BSA124S-CW, Sartorius), an ultrasonic instrument (PS-60AL, Shenzhen Redbon Electronics Co. Ltd.), a homogenizer (JXFSTPRP-24, Shanghai Jingxin Technology Co. Ltd.), a freeze dryer (LGJ-10C, Sihuan Fruike Instrument Technology Development Co. Ltd.), and a freeze centrifugal concentrator (CV600).

### 2.3 Nontargeted metabolomics analysis

#### 2.3.1 Metabolite extraction

Plasma samples were thawed at 4°C, and 50 µL of each sample was mixed with 200 µL of an extraction solution consisting of methanol and acetonitrile (1:1, v/v) containing deuterated internal standards (ISs). The mixture was vortexed for 30 seconds, sonicated for 10 minutes in a 4°C water bath, and incubated for 1 hour at -40°C. Subsequently, the samples were centrifuged at 12,000 rpm (relative centrifugal force: 13,800 × g; rotor radius: 8.6 cm) for 15 minutes at 4°C. The resulting supernatant was transferred to fresh glass vials for analysis. A quality control (QC) sample was prepared by pooling equal volumes of the supernatants. The supernatants were analyzed using ultra-high-performance liquid chromatography-tandem mass spectrometry (UHPLC-MS/MS).

#### 2.3.2 LC−MS/MS analysis

LC-MS/MS analyses were conducted using a Vanquish UHPLC system (Thermo Fisher Scientific) equipped with a Waters ACQUITY UPLC BEH Amide column (2.1 mm × 50 mm, 1.7 µm) and an Orbitrap Exploris 120 mass spectrometer (Thermo Fisher Scientific). The mobile phase comprised 25 mmol/L ammonium acetate and 25 mmol/L ammonium hydroxide in water (pH 9.75) as phase A, and acetonitrile as phase B. The autosampler temperature was maintained at 4°C, and the injection volume was set to 2 µL. The Orbitrap Exploris 120 operated in information-dependent acquisition (IDA) mode, controlled by Xcalibur software (Thermo), which continuously evaluated full-scan MS spectra. Electrospray ionization (ESI) source parameters included: sheath gas flow rate = 50 Arb, auxiliary gas flow rate = 15 Arb, capillary temperature = 320°C, full MS resolution = 60,000, MS/MS resolution = 15,000, collision energy = 20/30/40 stepped normalized collision energy (SNCE), and spray voltage = 3.8 kV (positive mode) or 3.4 kV (negative mode).

#### 2.3.3 Data preprocessing and annotation

The metabolomics raw data were converted to the mzXML format using ProteoWizard and processed with the R package XCMS (v3.5, CA, USA). Before data analysis, peak pretreatment steps included identification, alignment, extraction, and integration. Variability in the MS platform was monitored and adjusted using quality control (QC) spectra to ensure data reproducibility and reliability (QC details were shown in **Supplementary 1**).

In this study, 21,011 peaks were initially detected, and 17,333 peaks remained after relative standard deviation (RSD) de-noising. Missing values were imputed using half of the minimum value, and the internal standard normalization method was applied during data analysis. The final dataset, containing peak numbers, sample names, and normalized peak areas, was imported into the SIMCA 16.0.2 software package (Sartorius Stedim Data Analytics AB, Umeå, Sweden) for multivariate analysis. Data were scaled and log-transformed to reduce noise and the impact of high variable variance. Principal component analysis (PCA), an unsupervised dimensionality reduction method, was performed to visualize sample distribution and grouping. A 95% confidence interval in the PCA score plot was used to identify potential outliers and evaluate metabolic feature separation among groups.

To further investigate group separation and identify significantly altered metabolites, supervised orthogonal projections to latent structures-discriminant analysis (OPLS-DA) was applied. A 7-fold cross-validation was conducted to calculate R² and Q² values, where R² reflects the explained variation and Q² indicates predictive accuracy. Model robustness and predictive ability were assessed through a 200-time permutation test, which evaluated the likelihood of overfitting by examining the cross-validation-derived R² and Q² values (32, 33). A lower Q² intercept value indicated greater model reliability and reduced risk of overfitting.

The variable importance in projection (VIP) score of the first principal component in OPLS-DA summarized each variable’s contribution to the model. Metabolites with VIP > 1 and p < 0.05 (unpaired two-sided Student’s t-test) were identified as significantly altered. Remaining peaks were annotated by comparing retention time and mass-to-charge ratio (m/z) indices with the HMDB (www.hmdb.ca), KEGG (www.kegg.jp), and an in-house Biotree DB (V3.0) library (33, 34).

### 2.4 Targeted metabolomics analysis of amino acids

In total, 22 patients with MS and 22 CTR subjects from Yi and Han populations matched by age (± 3 years) and gender (1:1) were selected for targeted metabolomics analysis of amino acids. This analysis identified 71 endogenous amino acids.

#### 2.4.1 Metabolite extraction

After thawing the samples in an ice-water bath and vortexing for 30 seconds, 15 μL of each sample was combined with 35 μL of water and 200 μL of extraction solution (methanol: acetonitrile, 1:1 [v/v], containing deuterated internal standards, precooled to -40°C). The mixture was vortexed for 30 seconds, ultrasonicated for 15 minutes in a 4°C water bath, and incubated at −40°C for 1 hour. The samples were then centrifuged at 12,000 rpm (RCF = 13,800 × g, radius = 8.6 cm) for 15 minutes at 4°C. The supernatant was collected and evaporated to dryness. The dried residue was reconstituted in 100 μL of 50% methanol, followed by the addition of 100 μL of derivatizing agent and 50 μL of 1 M NaHCO_3_. After mixing, the sample was incubated at 40°C for 1 hour in a water bath. Once cooled to room temperature, 50 μL of 2 M HCl was added, and the sample was evaporated to dryness again. The final residue was re-dissolved in 200 μL of methanol and transferred to a fresh glass vial for analysis.

#### 2.4.2 Standard solution preparation

Stock solutions were prepared by dissolving or diluting each standard to a final concentration of 10 mmol/L. Aliquots of these solutions were combined in a 10-mL flask to create a mixed working standard solution. Absolute quantification was performed using isotope internal standard correction (**Supplementary Table S1**). Calibration standard solutions were prepared by serial dilution of the mixed working standard, ensuring isotopically labeled internal standards matched the sample concentrations.

#### 2.4.3 UHPLC-multiple reaction monitoring-MS analysis

Ultra-high-performance liquid chromatography (UHPLC) separation was performed on a Thermo Vanquish UHPLC System (Thermo Fisher) with a Waters ACQUITY UPLC BEH C18 column (100 mm × 2.1 mm, 1.7 μm). The mobile phases consisted of 5 mM ammonium acetate in water (A) and acetonitrile (B). The column temperature was maintained at 45°C, the autosampler at 4°C, and the injection volume was 2 μL.

Mass spectrometric analysis was conducted using a Thermo Altis TSQ Plus Mass Spectrometer (Thermo Fisher, USA) with an electrospray ionization (ESI) interface. Key ion source parameters included a spray voltage of -3300 V, sheath gas at 40 Arb, auxiliary gas at 10 Arb, sweep gas at 1 Arb, ion transfer tube temperature at 325°C, and vaporizer temperature at 350°C.

MRM parameters for targeted analytes were optimized using flow injection analysis. Standard solutions of individual analytes were injected into the mass spectrometer’s atmospheric pressure ionization (API) source. For each analyte, the most sensitive and selective Q1/Q3 transitions were designated as “quantifiers” for quantitative monitoring, while additional transitions were used as “qualifiers” to verify analyte identity.

**Supplementary Figure S1** presents the extracted ion chromatograms (EICs) for the targeted analytes from a standard solution (**Supplementary Figure S1A**) and a sample (**Supplementary Figure S1B**) under optimal conditions. The EICs demonstrate (i) symmetrical peak shapes for all analytes, (ii) baseline separations, and (iii) consistent retention times and peak shapes between the standard and the sample.

**Supplementary Table S2** details the lower limits of detection (LLODs) and quantitation (LLOQs) for all analytes. The LLODs ranged from 0.33 to 604.36 nmol/L, and the LLOQs ranged from 0.65 to 1208.72 nmol/L. Correlation coefficients (R²) for regression fitting exceeded 0.9933 for all analytes, confirming a robust quantitative relationship between MS responses and analyte concentrations, suitable for targeted metabolomics. **Supplementary Table S3** summarizes the analytical recoveries and relative standard deviations (RSDs) for quality control (QC) samples, measured over five technical replicates. Recoveries ranged from 81.0% to 109.0%, with RSDs below 6.6%, indicating that the method provides accurate quantitation of targeted metabolites within the specified concentration range.

#### 2.4.4 Data preprocessing and annotation

Calibration solutions were analyzed using UPLC-MRM-MS/MS methods as described. **Supplementary Table S10** outlines calibration curve parameters, where y represents the ratio of the analyte peak area to its corresponding internal standard, and x represents the analyte concentration (nmol/L). Regression fitting was performed using the least-squares method with 1/x weighting, which provided the highest accuracy and correlation coefficients (R²). Calibration levels were excluded if their accuracy fell outside the 80–120% range. Detailed calibration curves for individual analytes are provided in **Supplementary Table S10**.

Stepwise dilution of the calibration standard solution, with a dilution factor of 2, was performed for UHPLC-MRM-MS analysis. Signal-to-noise ratios (S/N) were used to define LLODs and LLOQs, corresponding to S/N values of 3 and 10, respectively, in accordance with US FDA guidelines for bioanalytical method validation.

Quantitation precision was assessed by calculating the RSD of replicate QC sample injections. Quantitation accuracy was determined as the analytical recovery of spiked QC samples, calculated as [(mean observed concentration)/(spiked concentration)] × 100%.

In the sample detection process, the final concentration (C*_F_*, nmol/L) equals the calculated concentration (C*_C_*, nmol/L) measured by the instrument, multiplied by the dilution factor. The concentration of the target metabolite (C*_M_*, nmol/L) in the sample is equal to the amount of C*_F_* times the final volume of the sample (V*_F_*, μ L), divided by the sample volume (V*_S_*, μ L), which is expressed as nmol/L. The following calculation formula was used:


$$c_{M}\left[\text{nmol}\cdot L^{-1}\right]=\frac{C_{F}\left[\text{nmol}\cdot L^{-1}\right]\cdot V_{F}\left[\text{μL}\right]}{\text{Vs}\left[\text{μL}\right]}$$

MRM data processing was performed using Skyline, while data acquisition utilized Xcalibur (version 4.4.16.14, Thermo Fisher). Metabolites with P<0.05 (unpaired two-sided Student’s t-tests) were identified as significantly altered. Pathway enrichment analysis of these metabolites was conducted using the KEGG database (http://www.genome.jp/kegg/).

### 2.5 Statistical analysis

Statistical analyses were performed using SPSS software (version 29.0, IBM). Continuous variables were assessed for normality with the Shapiro-Wilk test. Normally distributed variables are presented as mean ± standard deviation (SD) and compared using the Student’s t-test. Non-normally distributed variables are expressed as median (P_25_–P_75_) and analyzed with the Mann-Whitney U test. Categorical variables are reported as *n* (%) and compared using the χ² test. All statistical tests were two-tailed, with *P*<0.05 considered statistically significant.

## 3 Results

### 3.1 Characteristics of participants

The present study enrolled 84 participants, comprising 36 Yi individuals and 48 Han individuals, with no significant differences in age or sex between the two groups (*P*>0.05). The average age of the Han participants was 57.6 ± 7.9 years, while the Yi participants had an average age of 57.0 ± 11.9 years. MS patients in both populations exhibited higher body weight, BMI, total cholesterol (TC), LDL-C, TG, and uric acid (UA) levels compared to healthy counterparts (*P<*0.05). Among Yi subjects with MS, fasting plasma glucose (FPG), alanine transaminase (ALT), and heart rate were significantly elevated compared to healthy subjects (*P*<0.05). Conversely, no significant differences in FPG levels were observed between Han MS subjects and healthy control subjects (*P>*0.05). **Table 1** summarizes the demographic, anthropometric, and biochemical characteristics of the participants.

**Table 1:** Demographic data and biochemical index of the Yi and Han populations.

Variables	Han (n=48)				Yi (n=36)				*P-value*
	All (n=48)	HCTR (*n*=25)	HMS (n=23)	*P_h_-value*	All (n=36)	YCTR (n=21)	YMS (n=15)	*P_Y_-value*	
Age (years)	57.63 ± 7.92	57.48 ± 8.07	57.78 ± 7.94	0.90	57.06 ± 11.98	55.81 ± 12.54	58.8 ± 11.33	0.468	0.805
Female (%)	30 (62.5%)	17 (56.7%)	13 (43.3%)	0.412	24 (66.7%)	16 (66.7%)	8 (33.3%)	0.151	0.693
Smoking (%)	11 (22.9%)	6 (54.5%)	5 (45.5%)	1.00	6 (16.7%)	3 (50%)	3 (50%)	0.677	0.48
Drinking (%)	8 (16.7%)	4 (50%)	49 (50%)	1.00	9 (25%)	4 (44.4%)	5 (55.6%)	0.443	0.347
Height (m)	1.59 ± 0.08	1.59 ± 0.08	1.58 ± 0.08	0.87	1.59 ± 0.09	1.61 ± 0.08	1.56 ± 0.1	0.099	0.876
Weight (kg)	**62.08 ± 12.88**	**54.15 ± 7.2**	**70.69 ± 12.2**	**<0.001**	**58.93 ± 11.76**	**52.2 ± 5.92**	**68.36 ± 11.51**	**<0.001**	0.254
BMI (kg/m^2^)	**24.77 ± 5.34**	**21.48 ± 2.44**	**28.35 ± 5.37**	**<0.001**	**23.51 ± 5.15**	**20.19 ± 2.48**	**28.15 ± 4.24**	**<0.001**	0.278
SBP (mmHg)	**108.21 ± 31.26**	**98.03 ± 22.97**	**119.27 ± 35.59**	**0.02**	**131.93 ± 22.9**	**119.36 ± 13.56**	**149.53 ± 21.89**	**<0.001**	**<0.001**
DBP (mmHg)	**81.53 ± 13.95**	**74.12 ± 10.29**	**89.96 ± 12.87**	**<0.001**	**83.44 ± 15.24**	**74.91 ± 9.26**	**95.4 ± 13.99**	**<0.001**	0.554
Heart rate	59.06 ± 27.12	55.28 ± 25.36	63.17 ± 28.91	0.32	74.3 ± 12.15	72.9 ± 13.05	76.17 ± 10.98	0.439	**0.001**
AST (U/L)	26.68 ± 12.32	25.68 ± 12.77	27.77 ± 12	0.56	25.11 ± 8.94	24.25 ± 8.29	26.31 ± 9.94	0.504	0.518
ALT (U/L)	28.52 ± 19.2	23.7 ± 16.27	33.75 ± 21.07	0.07	**18.06 ± 5.92**	**14.55 ± 4.09**	**22.97 ± 4.41**	**<0.001**	**0.001**
TB (μmol/L)	13.98 ± 5.59	14.43 ± 6.1	13.5 ± 5.07	0.57	11.57 ± 4.96	12.08 ± 4.7	10.86 ± 5.38	0.473	**0.043**
IB (μmol/L)	9.73 ± 3.87	9.77 ± 4.21	9.69 ± 3.55	0.95	7.92 ± 3.66	8.16 ± 3.2	7.59 ± 4.31	0.648	**0.033**
UREA (mmol/L)	**5.72 ± 1.48**	**6.27 ± 1.44**	**5.12 ± 1.3**	**0.01**	5.06 ± 1.74	4.88 ± 1.58	5.31 ± 1.97	0.466	0.065
Cr (μmol/L)	69.39 ± 13.98	68.44 ± 14.69	70.41 ± 13.41	0.63	68.06 ± 13.56	64.33 ± 10.23	73.29 ± 16.12	0.071	0.665
TC (mmol/L)	**5.1 ± 0.92**	**4.56 ± 0.7**	**5.68 ± 0.78**	**<0.001**	**5.2 ± 1.05**	**4.51 ± 0.57**	**6.17 ± 0.75**	**<0.001**	0.62
LDL-C (mmol/L)	**3.05 ± 0.77**	**2.55 ± 0.46**	**3.59 ± 0.67**	**<0.001**	**2.88 ± 0.84**	**2.4 ± 0.48**	**3.56 ± 0.76**	**<0.002**	0.357
TG (mmol/L)	**2.16 ± 1.74**	**1 ± 0.3**	**3.43 ± 1.78**	**<0.001**	**1.85 ± 1.87**	**1.03 ± 0.35**	**3.01 ± 2.47**	**0.008**	0.439
HDL (mmol/L)	**1.35 ± 0.42**	**1.57 ± 0.41**	**1.11 ± 0.28**	**<0.001**	1.55 ± 0.42	1.66 ± 0.42	1.4 ± 0.37	0.061	**0.031**
UA (μmol/L)	**343.46 ± 101.21**	**301.41 ± 66.84**	**389.16 ± 113.23**	**<0.001**	**319.25 ± 129.98**	**263.91 ± 60.55**	**396.73 ± 161.15**	**0.007**	0.34
FPG (mmol/L)	**5.26 ± 2.07**	**4.28 ± 0.56**	**6.32 ± 2.55**	**<0.001**	**5.86 ± 3.1**	**4.67 ± 0.63**	**7.53 ± 4.28**	**0.022**	0.289
FT4 (pmol/L)	15.74 ± 2.24	15.59 ± 2.28	15.9 ± 2.24	0.63	13.89 ± 2.88	13.76 ± 2.83	14.08 ± 3.03	0.749	**0.001**
FT3 (pmol/L)	4.44 ± 0.52	4.39 ± 0.49	4.49 ± 0.56	0.51	4.33 ± 0.65	4.21 ± 0.49	4.51 ± 0.82	0.165	0.415
TSH (mIU/L)	2.8 ± 1.61	2.6 ± 1.55	3.02 ± 1.68	0.37	3.8 ± 4.81	4.05 ± 4.68	3.44 ± 5.15	0.712	0.239

BMI, body mass index; WC, waist circumference; SBP, systolic blood pressure; DBP, diastolic blood pressure; LDL, low density lipoprotein; TG, triglyceride; HDL, high density lipoprotein; FPG, fasting plasma glucose; CREA, serum creatinine; UA, uric acid; HCTR, Control of Han population; HMS, MS of Han population; YCTR, Control of Yi population; YMS, MS of Yi population; *P_h_-value*: HMS group versus HCTR; *P_Y_-value*: YMS group versus YCTR; *P-value:* Han population versus Yi population.

The bold index in the table refers to P<0.05 compared with MS groups.

### 3.2 Nontargeted metabolomics analysis

#### 3.2.1 Multivariate statistical analysis

**Figure 2:**
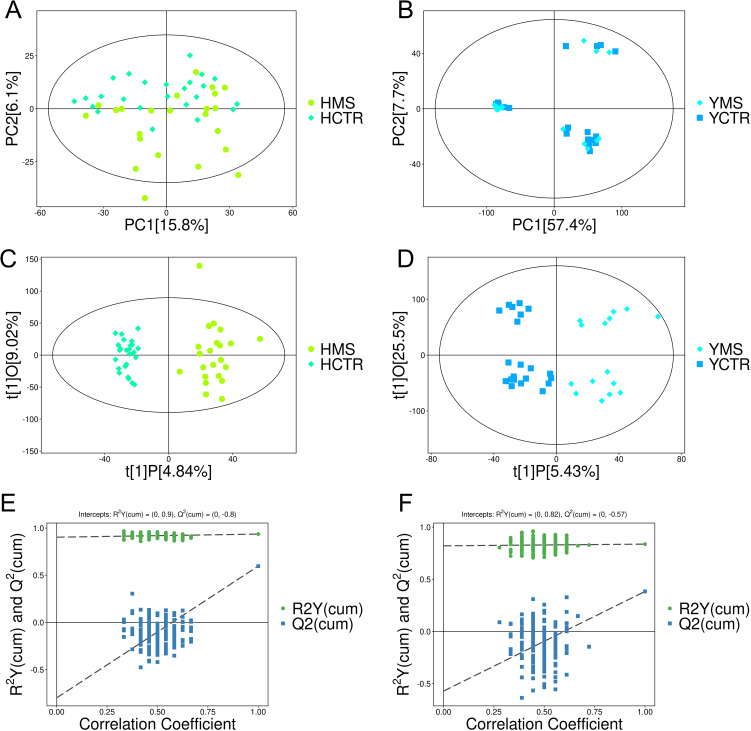
PCA and OPLS-DA score plots for the MS and CTR groups according to the nontargeted metabolic analysis. **(A, B)** PCA score plots for the MS and CTR groups in both populations. **(C, D)** OPLS-DA score plots for the MS and CTR groups in both populations. **(E, F)** Permutation tests for the OPLS-DA model.

A nontargeted LC-MS/MS metabolomics analysis was performed to investigate metabolic differences between the MS and CTR groups in the Yi and Han populations. PCA revealed significant distinctions between the CTR and MS groups within both ethnic groups (**Figures 2A, B**). OPLS-DA and its VIP scores identified key DEMs between the MS and CTR groups in both populations (**Figures 2C, D**). Similar to PCA, the OPLS-DA results demonstrated notable differences in metabolic profiles between the two groups. A permutation test confirmed the robustness of the OPLS-DA model, indicating no overfitting (P < 0.05). For the Han population, the R²Y and Q² intercepts were 0.9 and -0.8, respectively, while for the Yi population, these values were 0.82 and -0.57, respectively (**Figures 2E, F**). These results confirmed the high quality and reliability of the OPLS-DA model.

**Figure 3:**
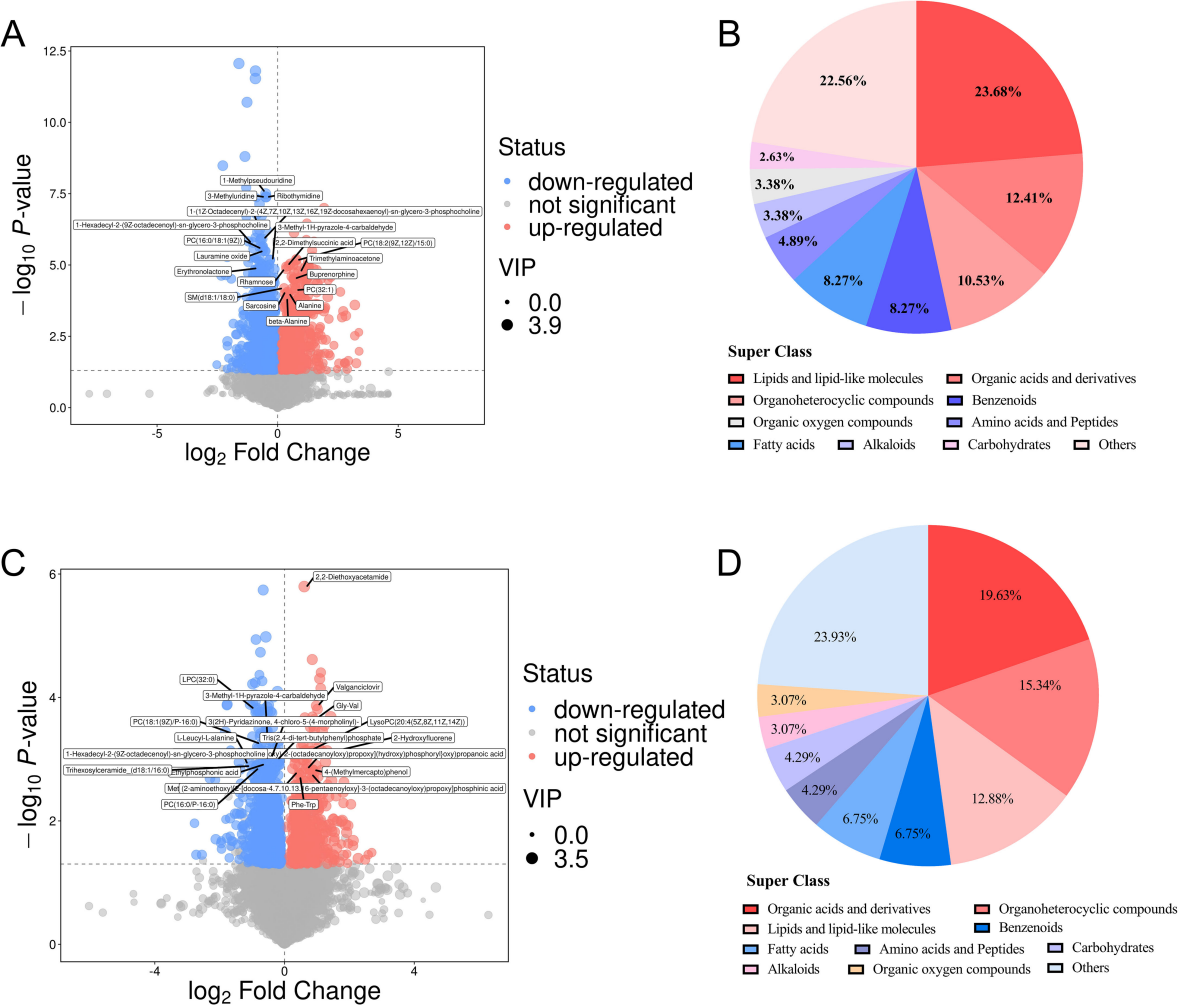
DEMs in the MS and CTR groups. **(A, C)** Volcano plot analysis of DEMS with VIP>1 and *P*<0.05 in both populations. **(B, D)** Distribution of all DEMs in both populations.

A total of 21,011 peaks were detected, and 17,333 metabolites were retained after de-noising based on relative standard deviation within the MS and CTR groups of the Han and Yi populations. Using the criteria VIP > 1 and P < 0.05, 2,762 and 1,535 significant DEMs were identified in the Han and Yi populations, respectively. In the Han population, 900 metabolites were upregulated and 1,862 were downregulated in the MS group (**Figure 3A**, **Supplementary Table S4**). Of these, 266 DEMs were matched using the KEGG and HDMB databases. The identified DEMs were categorized into 19 groups, with predominant classes including lipids and lipid-like molecules (23.68%), organic acids and derivatives (12.41%), organoheterocyclic compounds (10.53%), benzenoids (8.27%), and fatty acids (8.27%; **Figure 3B**). In the Yi population, 587 metabolites were upregulated and 948 were downregulated in the MS group (**Figure 3C**, **Supplementary Table S4**). Among these, 280 DEMs were matched using the KEGG and HDMB databases, with major categories comprising organic acids and derivatives (19.63%), organoheterocyclic compounds (15.34%), lipids and lipid-like molecules (12.88%), benzenoids (6.75%), and fatty acids (6.75%; **Figure 3D**).

#### 3.2.2 Common and unique DEMs of MS in the two populations

**Figure 4:**
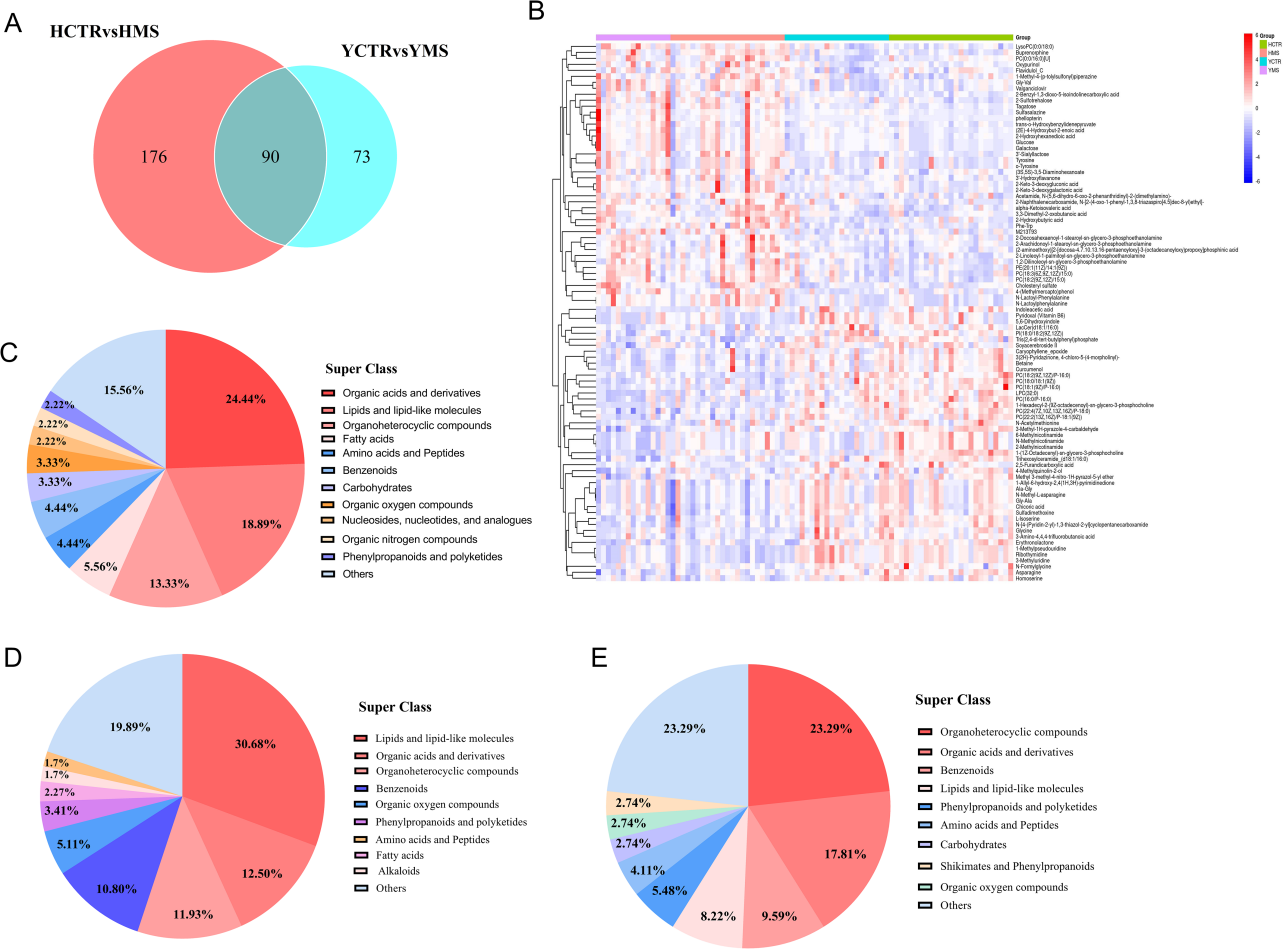
Analysis of DEMs in both populations. **(A)** Venn analysis in the MS group between the two populations. **(B)** Hierarchical cluster analysis heatmap of common DEMS in the MS groups in the two populations. **(C)** Distribution of common DEMs in the MS group of the two populations. **(D)** Distribution of DEMs only in the MS group in the Han Population. **(E)** Distribution of DEMs only in the MS group in the Yi Population.

Venn analysis was performed to explore the common and unique DEMs in the MS groups of the two populations. In total, 90 DEMs were changed in both MS groups of the two populations, including 46 upregulated metabolites and 44 downregulated metabolites (**Figures 4A, B**, **Supplementary Table S5**). These common DEMs were mainly organic acids and derivatives (24.44%), lipids and lipid-like molecules (18.89%), and organoheterocyclic compounds (13.33%) (**Figure 4C**).

In the Han population, there were 176 DEMs in the MS group, including lipids and lipid-like molecules (30.68%), organic acids and derivatives (12.5%), organoheterocyclic compounds (11.93%), and benzenoids (10.80%) (**Figure 4D**, **Supplementary Table S5**). In the Yi population, there were 73 DEMs, including organoheterocyclic compounds (23.29%), organic acids and derivatives (17.81%), benzenoids (9.59%), and lipids and lipid-like molecules (8.22%) (**Figure 4E**, **Supplementary Table S5**). Compared with the CTR groups, the serum metabolites in the MS groups in the two populations changed to varying degrees, with the three main metabolites that changed being organic acids and derivatives, lipids and lipid-like molecules, and organoheterocyclic compounds.

#### 3.2.3 Metabolic enrichment analysis and pathway analysis

**Figure 5:**
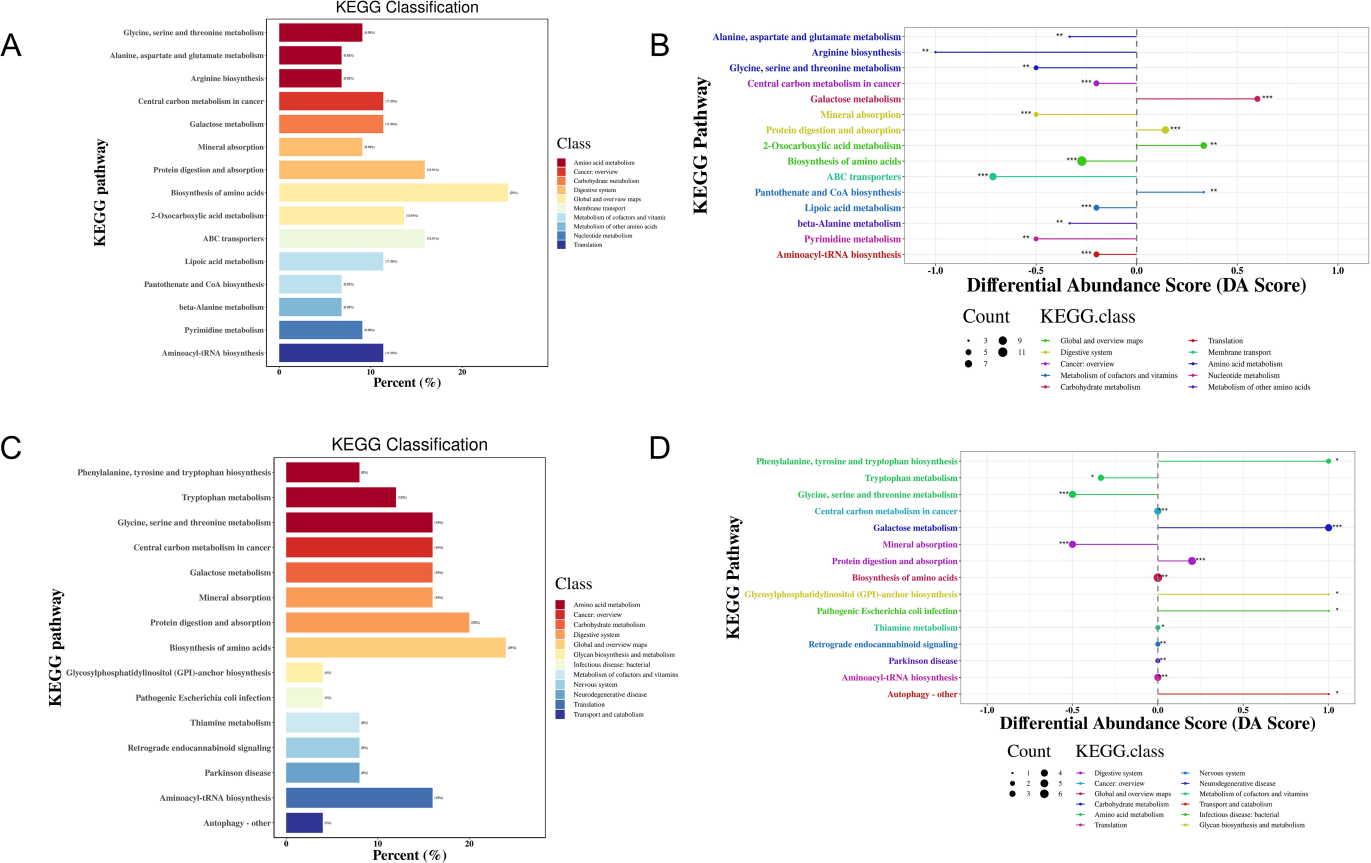
KEGG enrichment analysis of DEMs in the MS and CTR groups according to the nontargeted metabolic analysis. **(A, C)** Enrichment analysis of DEMs. **(B, D)** DA score analysis of DEMs. Each dot represents a metabolic pathway. The X-axis is the DA score, and the Y-axis is the ID number of the KEGG metabolic pathway. * represents significance (*P<0.05, **P<0.01, and ***P<0.001).

KEGG functional enrichment analysis of the metabolites indicated that 41 metabolic pathways may be involved in the pathogenesis of MS in the Han population. Most of these DEMs were predominantly enriched in amino acid synthesis and metabolism pathway (25%), followed by protein digestion and absorption (15.91%), ABC transporters (15.91%), and 2-oxocarboxylic acid (13.64%) (**Figure 5A**, **Supplementary Table S6**). Differential abundance (DA) score analysis was performed to determine the overall changes in all DEMs enriched in the same pathway. Galactose metabolism, protein digestion and absorption, 2-monocarboxylic acid metabolism, pantothenic acid metabolism, and CoA biosynthesis metabolism were significantly upregulated, while glycine, serine, and threonine metabolism, arginine synthesis metabolism, alanine, aspartic acid, and glutamic acid metabolism, cancer center carbon metabolism, mineral metabolism, amino acid synthesis metabolism, ABC transporter, thiamine metabolism, β-alanine metabolism, purine and pyrimidine metabolism, and amino acyl-tRNA biosynthesis metabolism were downregulated (**Figure 5B**, **Supplementary Table S6**).

For the Yi population, 35 metabolic pathways may be involved in the pathogenesis of MS. Most of these DEMs were predominantly enriched in amino acid synthesis and metabolism pathway (24%), followed by protein digestion and absorption (20%), biosynthesis of cofactors (20%), glycine, serine, and threonine metabolism, galactose metabolism, mineral metabolism, aminoacyl-tRNA biosynthesis, and cancer center carbon metabolism (16%) (**Figure 5C**, **Supplementary Table S6**). DA score analysis showed that pathways of phenylalanine and tryptophan synthesis metabolism, galactose metabolism, protein digestion and absorption, glycosylphosphatidylinositol anchor synthesis metabolism, pathogenic Escherichia coli infection, and autophagy were upregulated, while glycine, serine, and threonine metabolism, tryptophan metabolism, and mineral metabolism were downregulated (**Figure 5D**, **Supplementary Table S6**).

**Figure 6:**
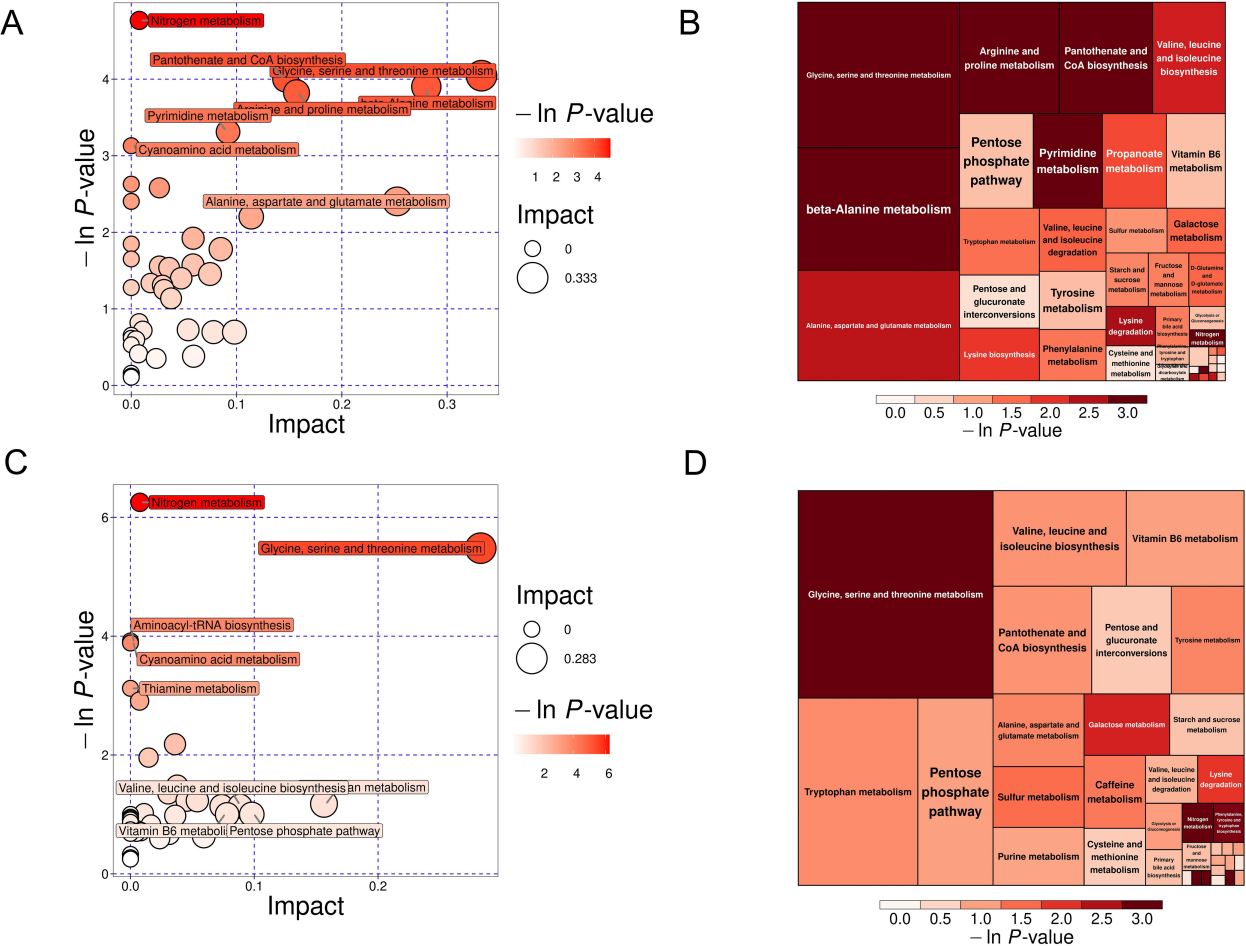
KEGG pathway analysis of DEMs in the MS and CTR groups. **(A, C)** Bubble plot analysis of pathways. Each related metabolic pathway is shown as a circle, in which the size and color are based on the pathway impact value and P-value, respectively. **(B, D)** Treemap plot analysis of pathways. Each related metabolic pathway is shown as a rectangle, in which the size and color are based on the pathway impact value and P-value, respectively.

Bioinformatics reduction and metabolic pathway enrichment analysis showed that seven pathways were significant (*P*<0.05) among the 39 metabolism pathways in the MS group in the Han population (**Figures 6A, B**, **Table 2**, **Supplementary Table S7**). Similarly, five pathways were significant (*P*<0.05) among the 35 metabolism pathways in the MS group in the Yi population (**Figures 6C, D**, **Table 3**, **Supplementary Table S7**). Metabolic pathways enrichment analysis in the different ethnic groups identified three common metabolic pathways (*P*<0.05) in both Yi and Han populations, including glycine, serine, and threonine metabolism, nitrogen metabolism, and cyanoamino acid metabolism, representing potential metabolic pathways for further research on MS in different ethnic groups.

**Table 2:** Seven differential metabolic pathways in the MS group of the Han population.

Pathway	Total	Hits	*P-value*	Impact	Hits DEMs
Glycine, serine, and threonine metabolism	48	4	0.017509	0.33266	Betaine; Glycine; Sarcosine; L-Homoserine
Beta-Alanine metabolism	28	3	0.020221	0.28022	Beta-Alanine; Uracil; 4-Aminobutyraldehyde;
Arginine and proline metabolism	77	5	0.021882	0.15694	L-Glutamine; Ornithine; Citrulline; Sarcosine; 4-Aminobutyraldehyde;
Pantothenate and CoA biosynthesis	27	3	0.018324	0.14652	Beta-Alanine; Alpha-ketoisovaleric acid; Uracil
Pyrimidine metabolism	60	4	0.036451	0.09193	L-Glutamine; Uridine; Uracil; Beta-Alanine
Nitrogen metabolism	39	4	0.0085119	0.00763	L-Tyrosine; L-Asparagine; L-Glutamine; Glycine;
Cyanoamino acid metabolism	16	2	0.04371	0	L-Asparagine; Glycine;

**Table 3:** Five differential metabolic pathways in the MS group of the Yi population.

Pathway	Total	Hits	*P-value*	Impact	Hits DEMs
Glycine, serine, and threonine metabolism	48	4	0.0041626	0.28293	Betaine; Glycine; L-Homoserine; L-Tryptophan;
Nitrogen metabolism	39	4	0.0019252	0.00763	L-Tyrosine; L-Tryptophan; L-Asparagine; Glycine;
Aminoacyl-tRNA biosynthesis	75	4	0.019865	0	L-Asparagine; Glycine; L-Tryptophan; L-Tyrosine;
Cyanoamino acid metabolism	16	2	0.020536	0	L-Asparagine; Glycine;
Thiamine metabolism	24	2	0.04403	0	L-Tyrosine; Glycine;

### 3.3 Targeted metabolomics analysis between the CTR and MS groups in the Yi and Han populations

Nontargeted metabolomics analysis revealed that the differential metabolic pathways between the MS and CTR groups, irrespective of ethnicity, predominantly involved amino acid synthesis pathways. To further investigate these findings, targeted amino acid metabolomics was performed to identify amino acid profiles significantly associated with MS in the Yi and Han populations of Yunnan Province.

#### 3.3.1 Multivariate statistical analysis

**Figure 7:**
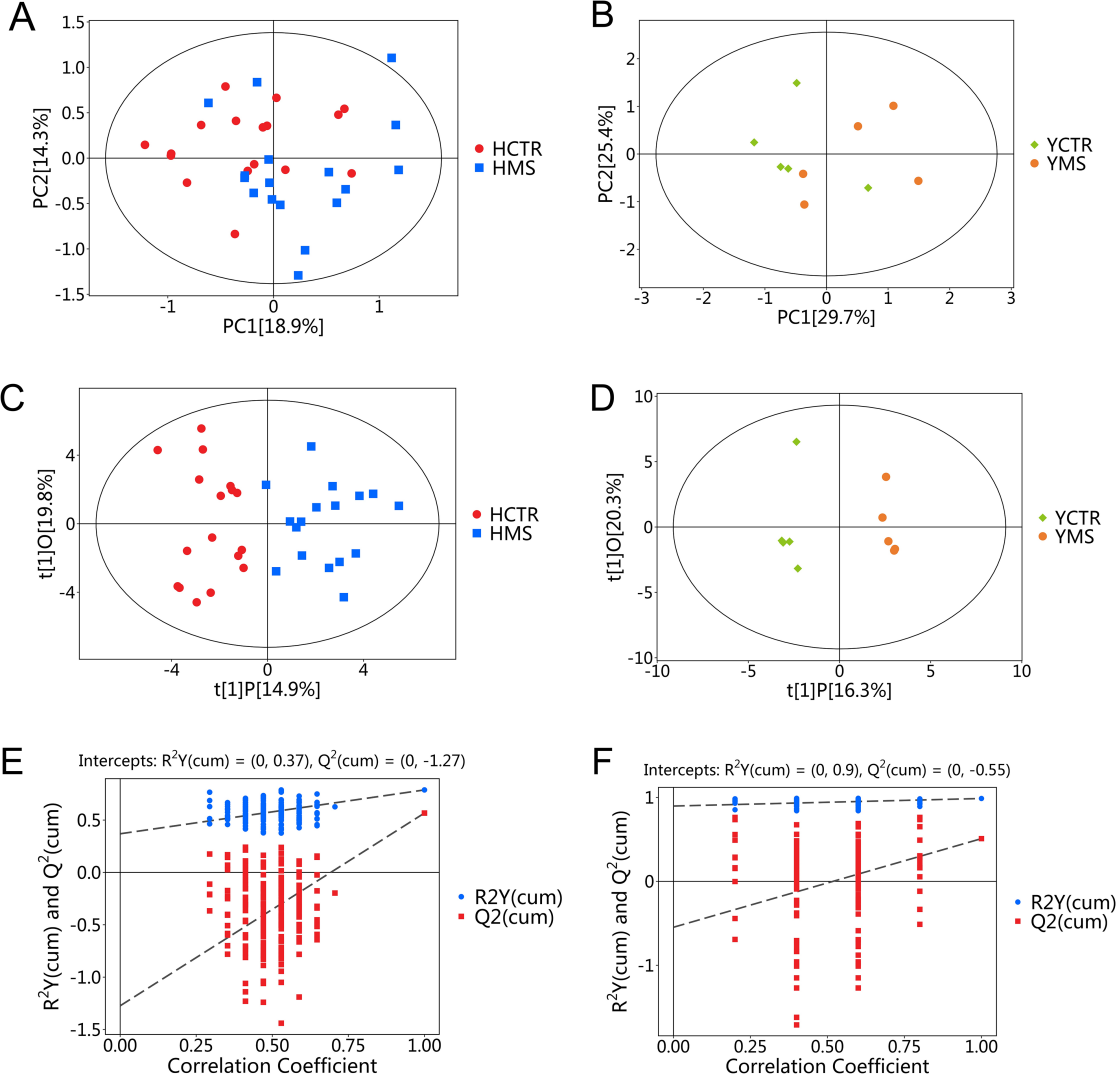
PCA and OPLS-DA score plots for the MS and CTR groups according to the targeted metabolic analysis. **(A, B)** PCA score plots for the MS and CTR groups in both populations. **(C, D)** OPLS-DA score plots for the MS and CTR groups in both populations. **(E, F)** Permutation tests for the OPLS-DA model.

PCA analysis further demonstrated clear separation between the CTR and MS groups in both populations (**Figures 7A, B**). Consistent with PCA results, the OPLS-DA model distinguished the MS and CTR groups effectively (**Figures 7C, D**). The permutation test again confirmed the model’s robustness, with no overfitting observed (*P* < 0.05). For the Han population, R²Y and Q² values were 0.37 and -1.27, respectively, while for the Yi population, these values were 0.9 and -0.55, respectively (**Figures 7E, F**). These results validated the reliability of the OPLS-DA model. Differential amino acid metabolites were identified between the CTR and MS groups in both populations.

**Figure 8:**
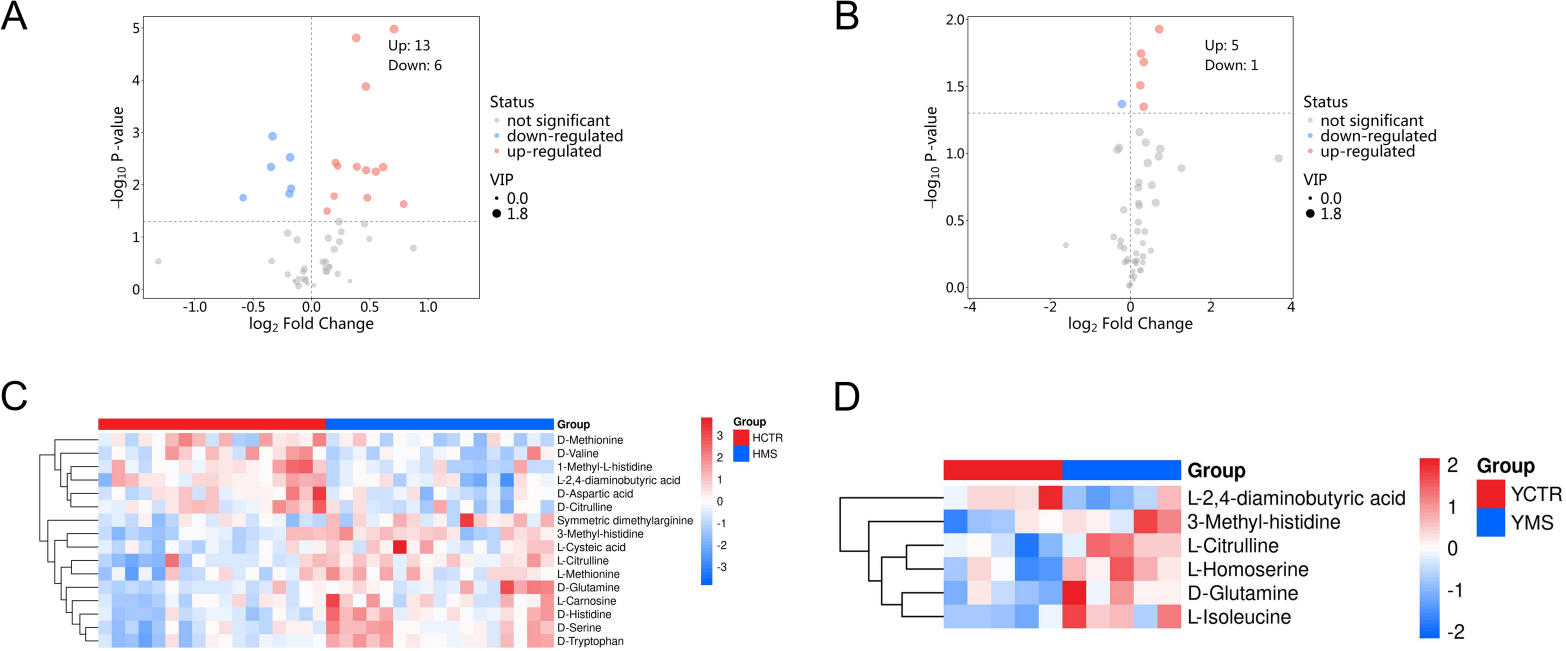
Differential amino acid metabolites in the MS and CTR groups. **(A, B)** Volcano plot analysis of DEMs with *P*<0.05 in both populations. **(C, D)** Hierarchical cluster analysis heatmap of the upregulated and downregulated differential amino acid metabolites in both populations.

A total of 71 amino acid metabolites were detected in plasma samples. We applied rigorous data management processes, retaining only metabolites with no more than 50% missing values in a single group or across all groups. Missing values were imputed using the minimum observed value multiplied by a random factor (0.1–0.5). After preprocessing, 52 metabolites were retained. Based on a screening criterion of P<0.05, 19 significantly altered amino acid metabolites were identified in the MS group of the Han population, including 13 upregulated and 6 downregulated metabolites. In the Yi population’s MS group, 6 significantly altered metabolites were identified, comprising 5 upregulated and 1 downregulated metabolite (P<0.05), as shown in **Tables 4** and **5**. The distribution of these differential amino acids is visualized through volcano plots and heatmaps for both populations (**Figures 8A–D**), highlighting metabolites such as D-glutamine, L-citrulline, and symmetric dimethylarginine (SDMA).

**Table 4:** Differential amino acids between HMS and HCTR by targeted metabolic analysis.

Compound name	HCTR	HMS	*VIP*	*P-value*	*Q-value*	FC
D-Histidine	101.30235	165.37735	1.78165	1.05339E-05	0.00040	1.63251
D-Tryptophan	151665.0767	197859.8625	1.77498	1.55337E-05	0.00040	1.30458
D-Serine	82690.20814	114246.5619	1.70320	0.00013	0.00229	1.38162
1-Methyl-L-histidine	245838.3513	195245.6494	1.71766	0.00118	0.01533	0.79420
L-2,4-diaminobutyric acid	893810.9686	787367.9074	1.72313	0.00299	0.02384	0.88091
L-Citrulline	132291.7693	152622.9844	1.26414	0.00378	0.02384	1.15368
3-Methyl-histidine	484299.2402	566218.7364	1.04375	0.00438	0.02384	1.16915
L-Cysteic acid	338.35029	443.07422	1.37148	0.00453	0.02384	1.30951
D-Citrulline	2862.56029	2251.235	1.51243	0.00455	0.02384	0.78644
L-Carnosine	1029.9879	1577.37128	1.66086	0.00458	0.02384	1.53145
Symmetric dimethylarginine	4246.02696	5877.41343	1.42537	0.00527	0.02426	1.38421
D-Glutamine	283865.3487	416106.9714	1.53709	0.00600	0.02426	1.46586
D-Valine	51029.92716	45227.29353	1.36884	0.01179	0.04716	0.88629
D-Aspartic acid	166151.0925	145853.6741	1.50783	0.01482	0.05416	0.87784
L-Methionine	49306.05255	56401.3001	0.99058	0.01649	0.05416	1.14390
D-Methionine	68.53971	45.69313	1.07876	0.01761	0.05416	0.66667
D-Threonine	432.19373	602.16059	1.22138	0.01771	0.05416	1.39327
D-Glutamic acid	423.88578	733.10020	1.18977	0.02335	0.06747	1.72948
Glutathione	44714.59716	49089.82069	1.07600	0.03186	0.08719	1.09785

**Table 5:** Differential amino acids between YMS and YCTR by targeted metabolic analysis.

Compound name	YCTR	YMS	VIP	*P-value*	*Q-value*	FC
3-Methyl-histidine	431876.5823	541785.3277	1.65865	0.04481	0.38839	1.25449
D-Glutamine	230295.1667	272940.3297	1.71560	0.03099	0.38839	1.18518
L-2,4-diaminobutyric acid	899996.084	776029.251	1.64883	0.04281	0.38839	0.86226
L-Citrulline	122926.7417	147458.6497	1.71998	0.01795	0.35996	1.19957
L-Homoserine	47183.37367	59302.66733	1.71992	0.02077	0.35996	1.25686
L-Isoleucine	3077.47533	5047.80833	1.82529	0.01180	0.35996	1.64024

#### 3.3.2 Association between metabolites and cardiometabolic risk factors

**Figure 9:**
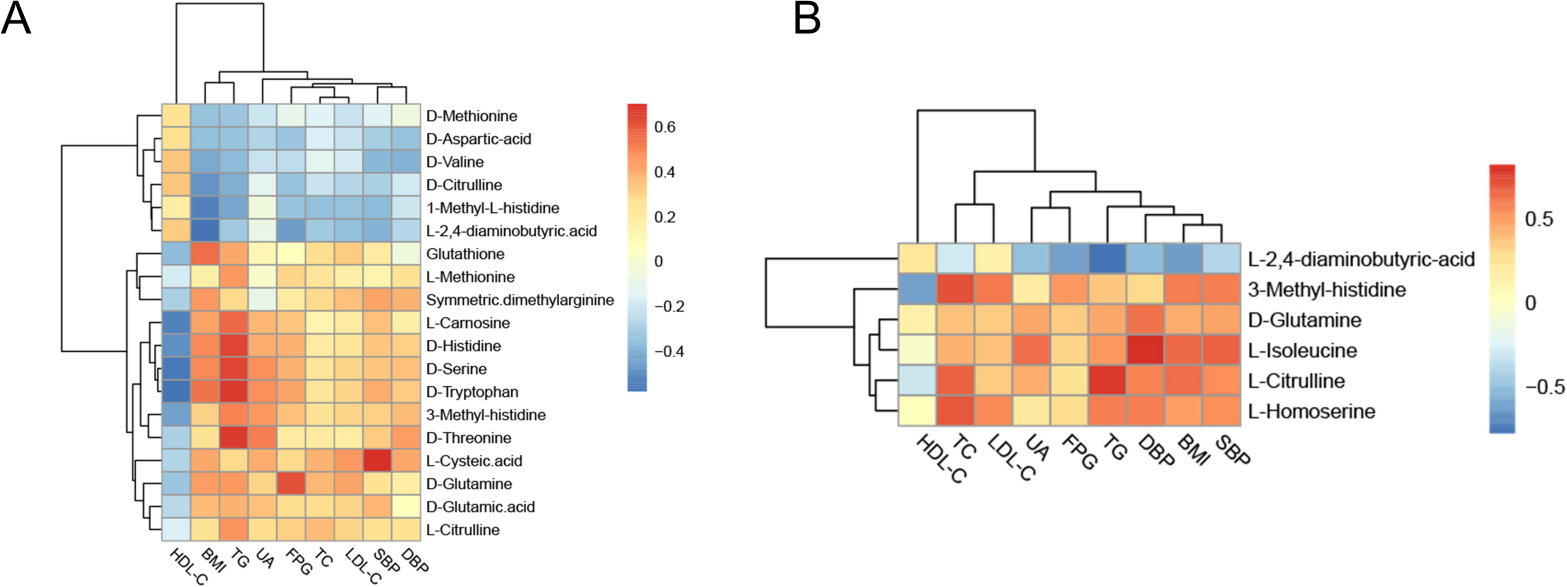
Correlation analysis of differential amino acids and metabolic components. **(A)** Heatmap of the correlation analysis in the Han population. **(B)** Heatmap of the correlation analysis in the Yi population. The color of each cell represents the Spearman’s correlation coefficient. Red indicates a positive correlation, and blue indicates a negative correlation.

To further clarify the mutual regulatory relationship between metabolites and MS, Spearman’s correlation analysis was performed to reveal the synergy of changes between metabolites and several cardiometabolic risk factors. 1-Methyl-L-histidine, D-aspartic acid, D-citrulline, D-methionine, D-valine, and L-2,4-aminobutyric acid were negatively correlated with BMI, BP, TC, TG, and LDL-C but positively correlated with HDL-C. The other differential amino acids were positively correlated with BMI, BP, TC, TG, and LDL-C but negatively correlated with HDL-C (**Figures 9A, B**, **Supplementary Table S8**).

#### 3.3.3 Metabolic enrichment analysis and pathway analysis of the CTR and MS groups in the Yi and Han populations

**Figure 10:**
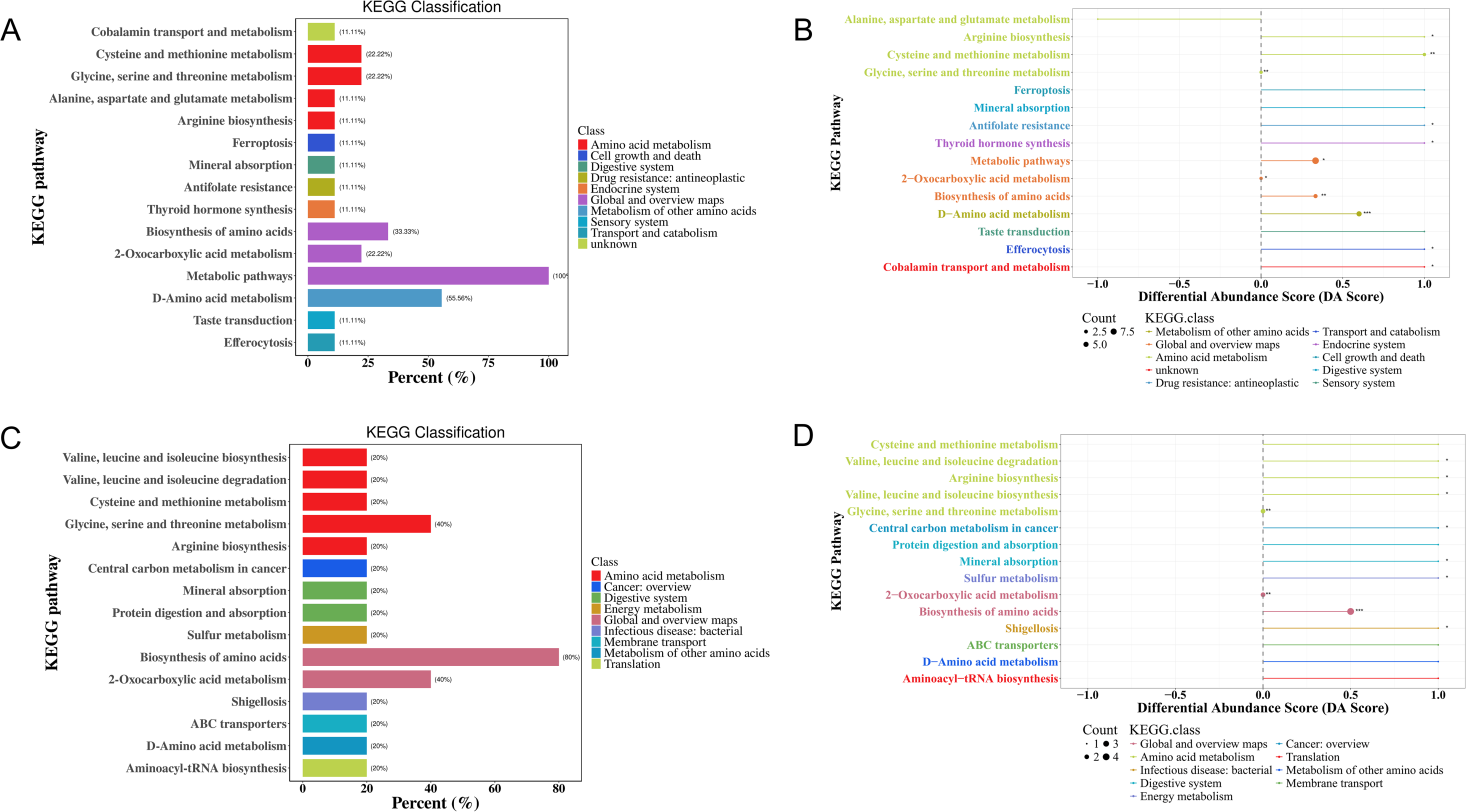
KEGG enrichment analysis of differential amino acid metabolites in the MS and CTR groups according to the nontargeted metabolic analysis. **(A, C)** Enrichment analysis of DEMs. **(B, D)** DA score analysis of amino acid metabolites. Each dot represents a metabolic pathway. The X-axis is the DA score, and the Y-axis is the ID number of the KEGG metabolic pathway. * represents significance (*P<0.05, **P<0.01, and ***P<0.001).

KEGG functional enrichment pathway analysis indicated that 25 metabolic pathways may be involved in the pathogenesis of MS in the Han population (**Supplementary Table S9**). Most of these amino acids were predominantly enriched in D-amino acid metabolism (55.56%), biosynthesis of amino acids (33.33%), 2-oxocarboxylic acid metabolism, biosynthesis of cofactors, glycine, serine, and threonine metabolism, and cysteine and methionine metabolism (22.22%) (**Figure 10A**, **Supplementary Table S9**). DA score analysis showed that alanine, aspartate, and glutamate metabolism was significantly downregulated, while the other pathways were upregulated, except for 2-oxocarboxylic acid metabolism and glycine, serine, and threonine metabolism (**Figure 10B**, **Supplementary Table S9**).

For the Yi population, KEGG pathway analysis identified the involvement of 16 metabolic pathways in the pathogenesis of MS. Most of these amino acids were predominantly enriched in the biosynthesis of amino acids (80%), 2-oxocarboxylic acid metabolism (40%), glycine, serine, and threonine metabolism (40%), and the other metabolic pathways (20%) (**Figure 10C**). DA score analysis showed that all pathways were significantly upregulated, except for 2-oxocarboxylic acid metabolism, glycine, serine, and threonine metabolism (**Figure 10D**, **Supplementary Table S9** **).**

**Figure 11:**
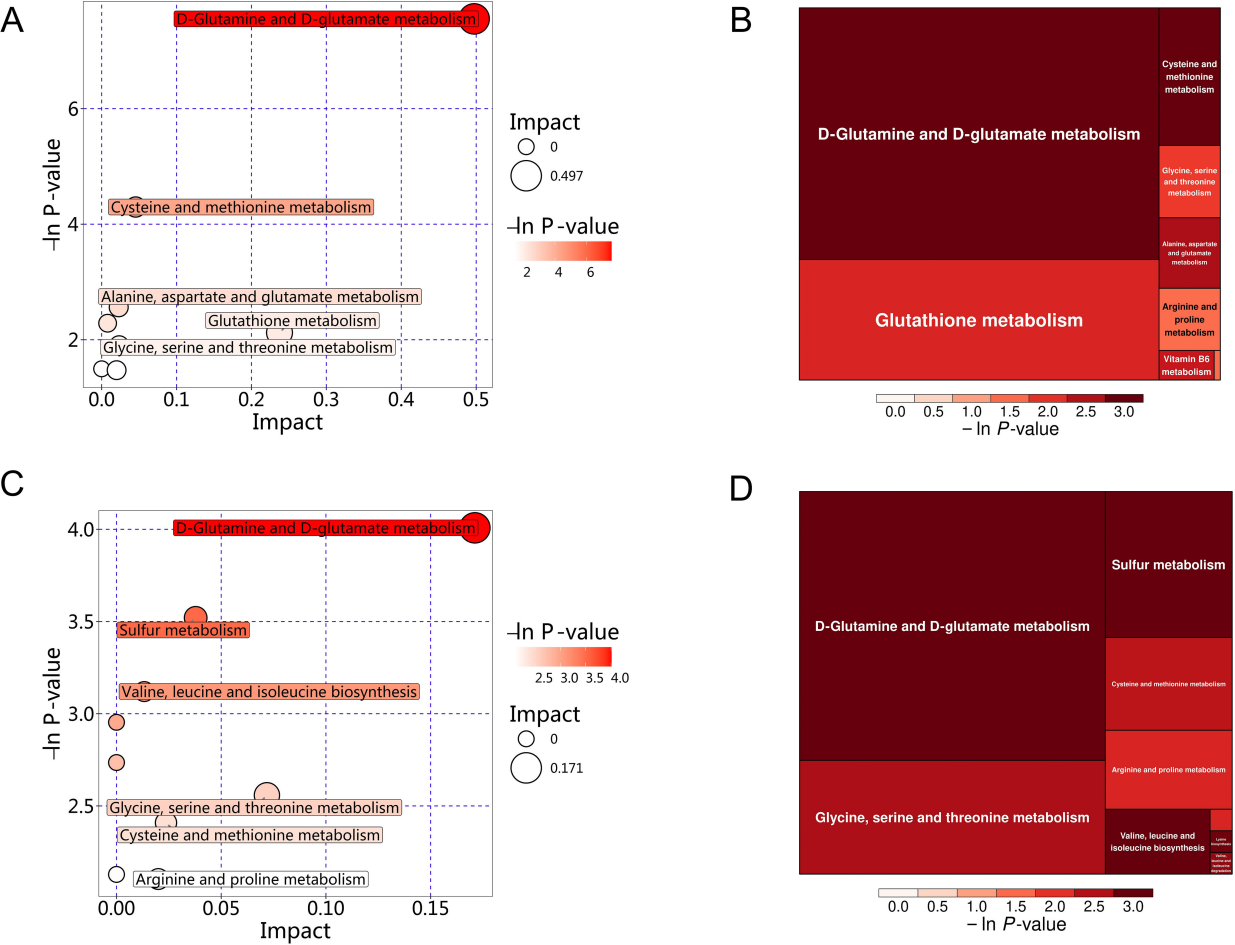
KEGG pathway analysis of differential amino acid metabolites in the MS and CTR groups. **(A, C)** Bubble plot analysis of pathways. Each related metabolic pathway is shown as a circle, in which the size and color are based on the pathway impact value and *P*-value, respectively. **(B, D)** Treemap plot analysis of pathways. Each related metabolic pathway is shown as a rectangle, in which the size and color are based on the pathway impact value and *P*-value, respectively.

The significant differential amino acid metabolites were enriched to further analyze the metabolomic pathways involved in MS. There were two significant pathways (*P*<0.05) among the 25 metabolism pathways in the MS group in Han population, including D-glutamine and D-glutamate metabolism, as well as cysteine and methionine metabolism, as shown in **Table 6** and **Figures 11A, B**. For the Yi population, there were three significant pathways (P<0.05) among the 16 metabolic pathways, namely, namely, D-glutamine and D-glutamate metabolism, sulfur metabolism, and valine, leucine, and isoleucine biosynthesis (**Figures 11C, D**, **Table 6**). Notably, D-glutamine and D-glutamate metabolism was significantly enriched in the MS group of both populations, suggesting that these targets should be further explored to investigate the pathological mechanism underlying MS.

**Table 6:** Significant pathways between MS and CTR by targeted metabolic analysis in two distinct populations.

Ethnic	Pathway	Total	Hits	*P*-value	Impact	Hits DEMs
Han population	D-Glutamine and D-glutamate metabolism	11	2	0.00052	0.49732	D-Glutamine; D-Glutamic acid
	Cysteine and methionine metabolism	56	2	0.01361	0.04541	L-Methionine; Glutathione;
Yi population	D-Glutamine and D-glutamate metabolism	11	1	0.01817	0.17112	D-Glutamine
	Sulfur metabolism	18	1	0.02960	0.0378	L-Homoserine
	Valine, leucine and isoleucine biosynthesis	27	1	0.044147	0.01325	L-Isoleucine

## 4 Discussion

As a cluster of cardiac metabolic risk factors, the pathogenesis of MS remains unclear. It is associated with genetic predisposition, lifestyle, diet, and other contributing factors. The epidemiological characteristics of MS may be different among people with different eating habits and genetic backgrounds (8, 18, 20). Amino acids are essential components of the human body, as protein precursors, amino acids participate in various physiological activities and metabolism of the human body and play an important role in the progression of many diseases (35). In the early stage of metabolic disease, the imbalance of metabolic activity in the body may lead to changes in the levels of some specific amino acids, and in-depth study of MS metabolites in different ethnic groups. Identifying similarities as well as differences in associations between alterations in the serum metabolome and metabolic syndrome across ethnic groups is helpful for us to understand the commonness and characteristics of the disease, which could provide insights for the effective prevention and treatment of MS in various populations.

Identifying common DEMs and metabolic pathways across populations enhances our understanding of disease mechanisms under differing genetic backgrounds, offering a foundation for novel clues in preventing and managing risk factors such as obesity, hypertension, dyslipidemia, and glucose abnormalities in diverse populations. In our study, nontargeted metabolomics analysis identified three common metabolic pathways in both Yi and Han populations, including glycine, serine, and threonine metabolism, nitrogen metabolism, and cyanoamino acid metabolism. Betaine, glycine, L-Homoserine and L-Asparagine are several differential metabolites the above metabolic pathways hit and all showing decreased levels in MS groups across both ethnic groups. Betaine is the source of methyl donor, which provides methyl to convert Hcy to methionine, reducing the accumulation of HCY and the availability of fatty acid substrate (acetyl CoA) (36). Previous studies have shown that plasma betaine is negatively correlated with body mass index (BMI), body fat percentage and waist circumference (37). Betaine could also activate AMPK pathway, regulating the expression of PPARα and its downstream fatty acid oxidation related genes, thereby increasing the activity of carnitine palmitoyl transferase 1, promoting the β oxidation of atty acids in mitochondria, negatively regulating fat synthesis, and improving lipid accumulation and islet resistance in HFD-induced obese mice (38). Abnormal metabolism of glycine, serine, and threonine is closely associated with obesity, insulin resistance, and various cardiac metabolic diseases (39, 40). Glycine is primarily synthesized in the liver and kidney from choline, oxalate, betaine, and glucose (10), contributes to the production of creatine, glutathione, purines, glucose, and collagen. Circulating glycine deficiency has been implicated in metabolic dysfunction-related fatty liver disease, atherosclerosis, myocardial infarction, and MS (41, 42). Under insulin-resistant conditions, hepatocytes prioritize glycine and serine for glucose synthesis, leading to decreased circulating levels of these amino acids (22, 39). Additionally, glutathione synthesis is upregulated in response to oxidative stress during insulin resistance, further depleting glycine and serine (43). In obesity, serine serves as a precursor for sphingolipid synthesis, promoting the accumulation of bioactive lipid ceramides in insulin-sensitive tissues such as the liver and muscle, thereby exacerbating insulin resistance (39). In targeted metabolomics analysis, D-glutamine and D-glutamate metabolism emerged as a significantly enriched pathway in both groups, and D-glutamine, L-citrulline, SDMA, and L-2,4-diaminobutyric acid were common differential amino acids. Glutamine is vital in glutathione production, redox homeostasis, and intracellular acid-base balance (44). Following the conversion of glutamate to α-KG, α-KG enters the tricarboxylic acid (TCA) cycle and serves as a substrate for intracellular glutathione (GSH) synthesis and turnover. GSH is further converted to ammonia by γ-glutamyl transpeptidase (GGT) and recycled as glutamate (45). Under pathological conditions associated with insulin resistance, such as non-alcoholic fatty liver disease (NAFLD), obesity, and MS, altered mitochondrial metabolism and increased energy demand in the liver elevate transaminase levels and glutamate release (46, 47). Serum glutamine levels in the prediabetic MS group are 4.8 times higher than those in the control group, while glutamine levels in MS group patients with normal blood glucose are 3.5 times higher than those in control group subjects, underscoring the connection between glutamine, blood glucose, and MS (48). D-glutamine and D-glutamate metabolism, arginine biosynthesis, glutathione metabolism, and phenylalanine and lysine degradation pathways have been reported to be significantly affected in MS (48). Compared with non-progressive individuals, those who progress to pre-T2D within 5 years exhibit higher baseline levels of four polar amino acids (aspartic acid, asparagine, glutamine, and glutamic acid) and one aromatic amino acid (phenylalanine), which are critical predictors of T2D (49).

Furthermore, focusing on the unique metabolites and pathways associated with metabolic syndrome in individuals from different living environments and genetic backgrounds, may offer insights for personalized prevention and treatment of metabolic syndrome across diverse ethnic groups. In targeted metabonomic analysis, Sulfur metabolism, Cysteine and methionine metabolism and Valine, leucine and isoleucine biosynthesis changed significantly in MS of Yi nationality, L-tryptophan and L-Isoleucine were higher than those in the control group of Yi population. Increased levels of plasma BCAAs (leucine, isoleucine, and valine) and AAAs (phenylalanine, tyrosine, and tryptophan) are associated with visceral obesity, insulin resistance, glucose metabolism, lipid metabolism, diabetes, and CVD (11, 50). As direct activators of mammalian target of rapamycin (mTOR) signal transduction, BCAAs activate the mTORC protein kinase to enhance phosphorylation of insulin receptor substrate-1 on inhibitory sites, leading to blunted phosphatidylinositol-3-kinase activation and downstream insulin signaling (42). The plasma levels of BCAAs in T2D patients with poor blood glucose control are approximately 40% higher than those in controls (51), and the levels of these three amino acids are positively correlated with the levels of FPG and TG (50). There is a significant change in Cysteine and methionine metabolism in Han MS, which is closely related to the synthesis of GSH. Glutathione levels were markedly increased in the MS group of the Han 464 population, GSH scavenges ROS and is reversibly oxidized to oxidized glutathione (GSSG), which plays an important role in the process of antagonizing oxidative stress, while oxidative stress is closely related to metabolic syndrome (55, 56). At the same time, we found that the levels of D-threonine, D-serine, L-Methionine and Glutamic acid in the MS group were also significantly higher than those in the CTR group in the Han population were found in MS of Han nationality, and the above metabolites were closely related to the metabolism of GSH (55, 57). These changes may result from mitochondrial dysfunction and altered metabolism in MS, which elevates glutamate levels, while oxidative stress intensifies the demand for GSH. These findings align with those of previous reports (11, 43, 48). In addition, the levels of D-Tryptophan, histidine, valine and other amino acids also changed in the MS group of Han population (**Table 4**). Tryptophan is composed of β-carbon connected to an indole group, and it is catabolized in the proinflammatory state to produce various signal substances through the canine uric acid, hydroxycanine, indole, and 5-hydroxytryptamine pathways (52, 53). The serum levels of tryptophan and tyrosine in MS patients with prediabetes are significantly higher than those in healthy controls (48). The serum levels of tryptophan and its two downstream products (canine uric acid and xanthurenic acid) are increased in patients with MS, and the ratio of tryptophan to canine uric acid is higher (54). High levels of valine also produce the 3-hydroxyisobutyrate (3-HIB) metabolite, which can activate the transport of fatty acids across endothelial cells, resulting in local tissue lipid accumulation and lipotoxicity, ultimately compromising insulin signaling (42, 55). Arginine is the precursor of urea, polyamine, proline, nitric oxide, creatine, glutamic acid, and agmatine (56). symmetric dimethylarginine (SDMA)is a derivative of L-arginine generated by the posttranslational methylation of arginine residues, such as its isomer, and SDMA may interfere with the use of the L-arginine enzyme substrate, which diminishes NO bioavailability (53). The increase of free 1-methylhistidine is associated with higher SBP and DBP in African Americans (57). Histidine is an essential amino acid that has been demonstrated to have antioxidant properties, reduce inflammatory burden, and reduce oxidative stress, and it is closely related to insulin sensitivity, obesity, and MS (58, 59). Histidine supplementation has been found to improve insulin resistance, blood lipid levels and inflammation, and delay the development of atherosclerosis in rodent models of diabetes and metabolic syndrome (60).

Oxidative stress is closely related to the occurrence of metabolic syndrome (61). GSH production is a key defense against oxidative stress, but its balance is frequently disrupted in metabolic diseases (19). In our study, serum metabolomics analysis of MS in two distinct ethnic groups highlights the pivotal role of amino acid metabolic disorders in MS pathogenesis. Notably, no matter the glycine-serine-threonine pathway, significantly enriched in non-targeted metabolomics, or the D-glutamine and D-glutamate pathway, cysteine and methionine metabolism identified in targeted metabolomics, are closely linked to glutathione synthesis, which suggested that the imbalance of oxidative stress may be a common and potential driver of MS across ethnic groups. However, as our study was cross-sectional with a limited sample size, the causal relationship between serum amino acids and the risk of MS cannot be clarified, further research is required to validate the roles of these DEMs and pathways in MS.

## 5 Conclusions

Our study investigates metabolic differences in metabolic syndrome (MS) between Yi and Han populations through nontargeted and targeted metabolomics approaches, identifying both common and unique metabolites and metabolic pathways associated with MS, especially amino acid metabolic disorders, including glycine, serine, and threonine metabolism, D-glutamine and D-glutamate metabolism, which may play critical roles in regulating different metabolic dysfunctions and worth further exploration in MS pathogenesis, which might provide insights for the effective prevention and treatment of MS in various populations.

## Data Availability

The original contributions presented in the study are included in the article/**Supplementary Material**. Further inquiries can be directed to the corresponding authors.
