# Supplementary material for: Nontargeted and targeted metabolic profile of metabolic syndrome patients: a study based on Yi and Han populations in Yunnan
Source: Front Endocrinol (Lausanne). 2025 May 14;16:1488099. doi: 10.3389/fendo.2025.1488099 (PMC12116332; doi:10.3389/fendo.2025.1488099)
Supplement: Supplementary file 1 [file DataSheet1.zip › 1488099_SupMaterial/Data Sheet 2.PDF]

Charlesworth Author Services

EDITORIAL CERTIFICATE

This document certifies that the manuscript below was edited for correct English language usage, grammar, punctuation and spelling by qualified native English speaking editors at Charlesworth Author Services.

Paper Title:

Nontargeted and Targeted Metabolic Profile of Metabolic Syndrome Patients: A study based on Yi and Han Populations in Yunnan

Author:

Yan-Mei Ji

Date certificate issued:

December 27, 2024

cwauthors.com
