# Supplementary material for: Nontargeted and targeted metabolic profile of metabolic syndrome patients: a study based on Yi and Han populations in Yunnan
Source: Front Endocrinol (Lausanne). 2025 May 14;16:1488099. doi: 10.3389/fendo.2025.1488099 (PMC12116332; doi:10.3389/fendo.2025.1488099)
Supplement: Supplementary file 1 [file DataSheet1.zip › 1488099_SupMaterial/Supplement 1-QC signals.pdf]

1. TIC diagram of Experimental QC sample

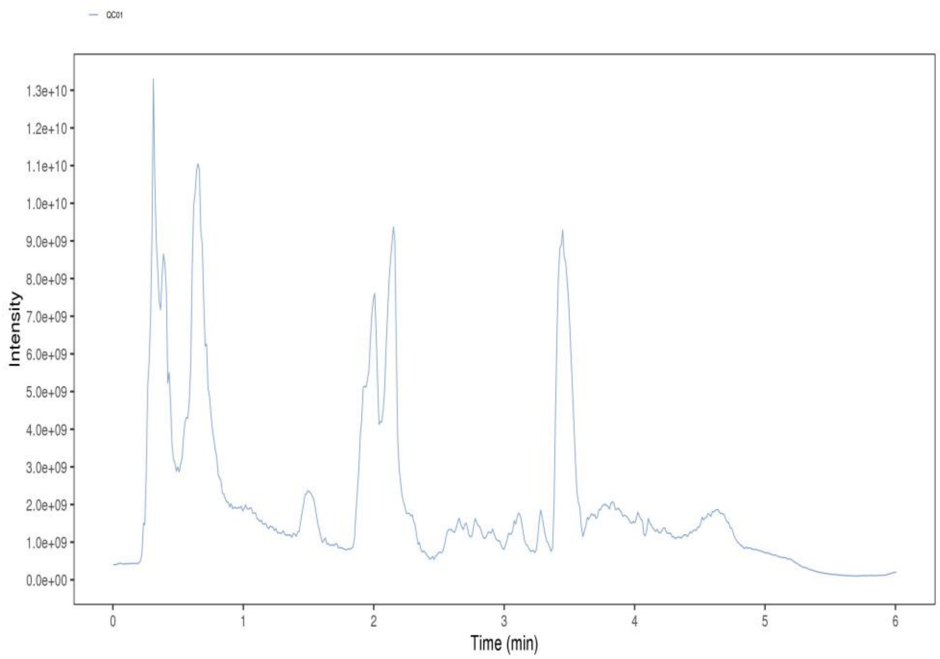

Figure S1. TIC diagram of QC samples detected by UHPLC-OE-MS in Positive ion mode

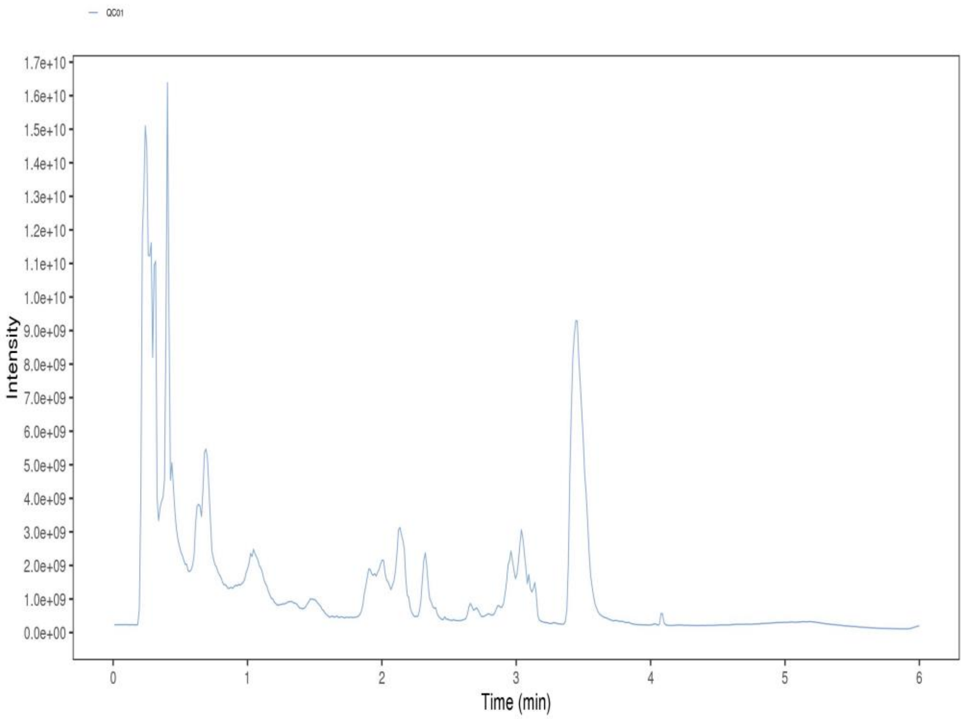

Figure S2. TIC diagram of QC samples detected by UHPLC-OE-MS in negative ion mode

2. Quality Control

2.1 Peak height difference of internal standard between QC samples

The stability of the test can be judged by the difference of the response peak height of the internal standard among the QC samples. From Figure S3 and S4, we can see that the retention time and response strength of the internal standard in the QC samples are very stable. It shows that the data acquisition stability of the instrument is very good.

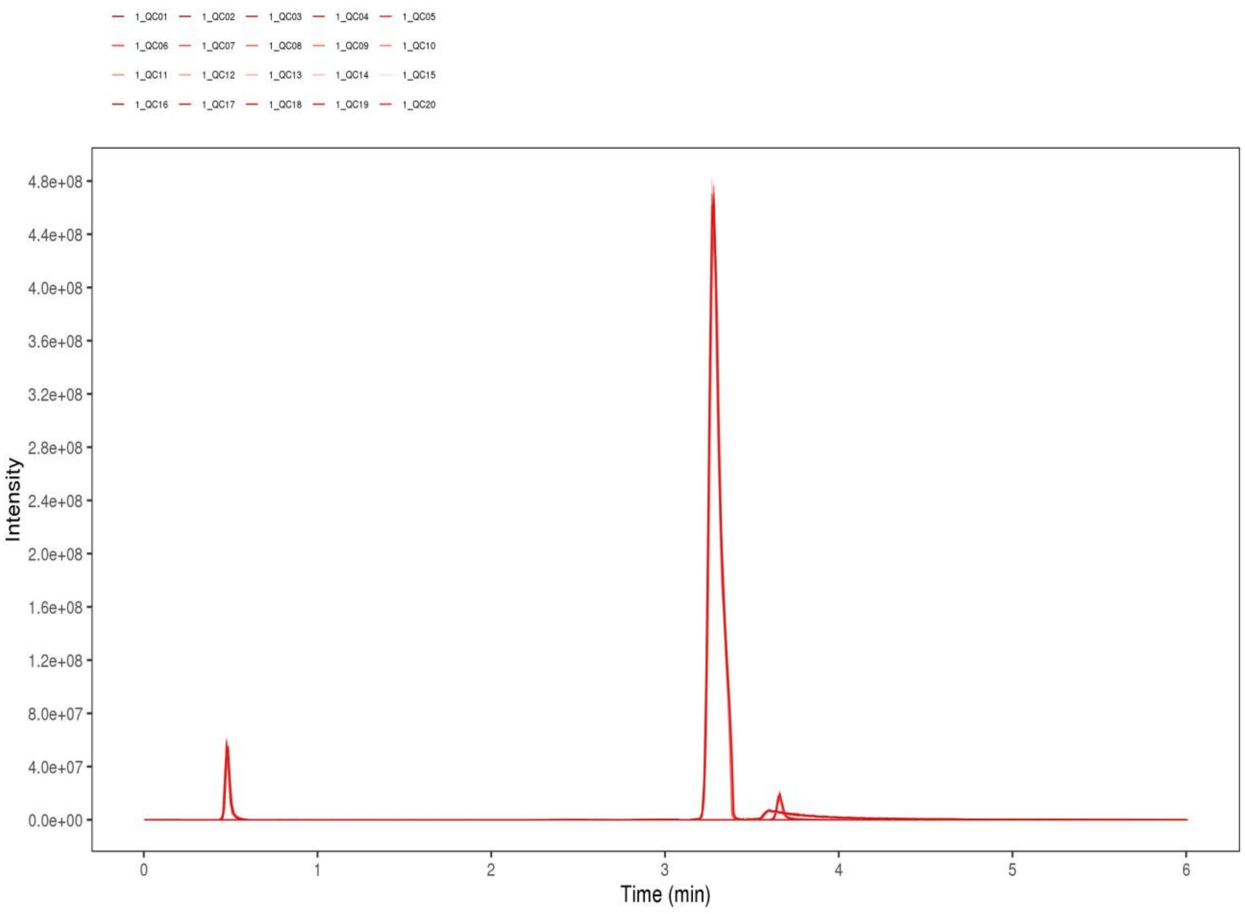

Figure S3. EIC diagrams of internal standard positive ions in all QC samples.

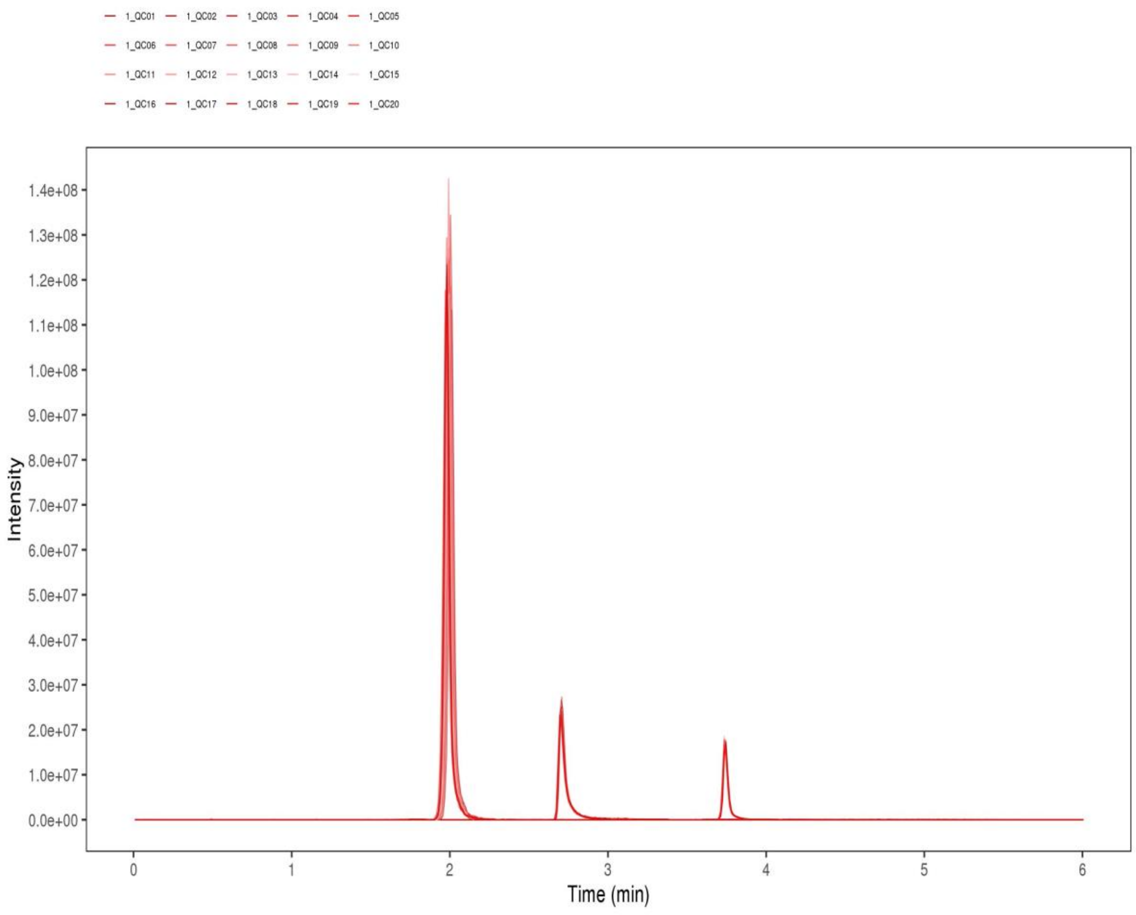

Figure S4. EIC diagrams of internal standard negative ions in all QC samples

2.2 Peak appearance of internal standard in blank sample

Through the detection of the blank samples interspersed in the whole process of the experiment, the residue of the substance in the detection process can be investigated. From Figure S5 and Figure S6, we can see that all the internal standards in all blank samples have no obvious peaks detected, indicating that the substance residues are well controlled and the cross-contamination between samples is controlled in a controllable range.

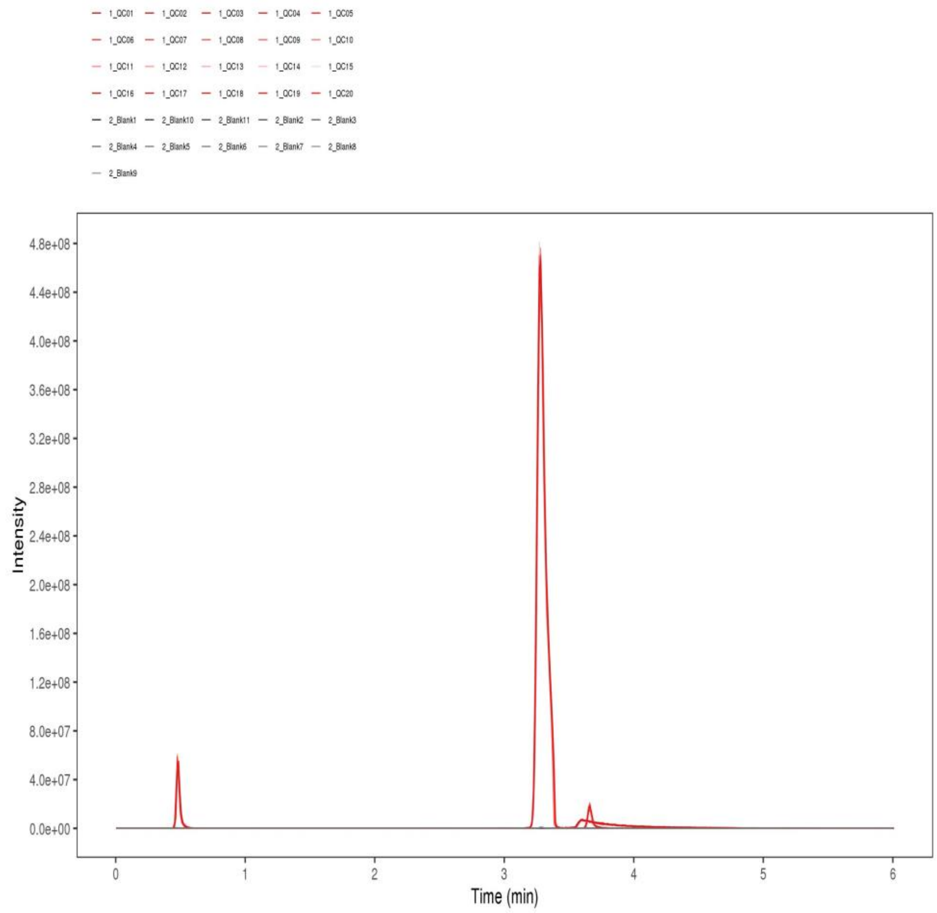

Figure S5. Internal standard positive ion EIC diagram of blank sample and QC sample

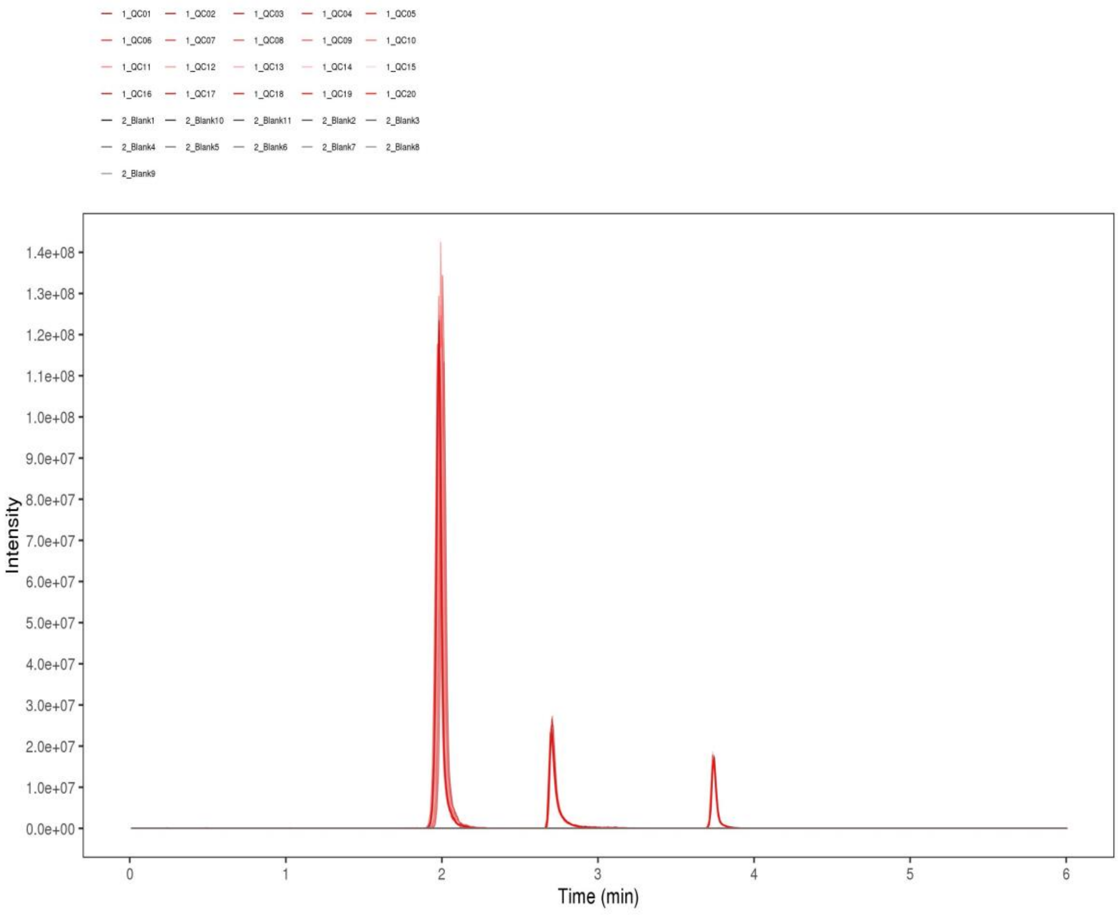

Figure S6. Internal standard negative ion EIC diagram of blank sample and QC sample

3. Data quality control

3.1 Presentation of QC samples in two-dimensional PCA score Chart

It can be seen from Figure S7 that the aggregation of QC samples is very good, indicating that the method is stable.

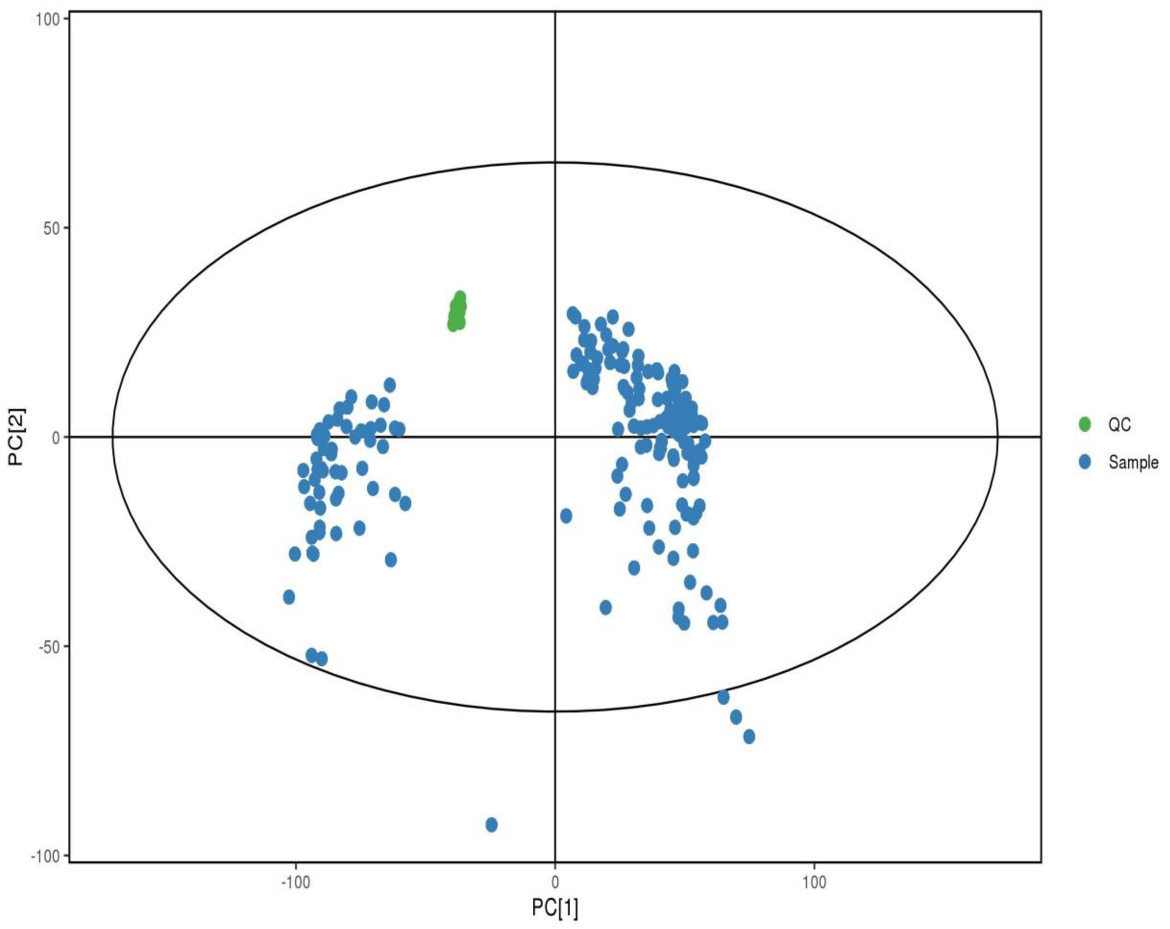

Figure S7 . PCA score chart. The green refers to the QC sample, and the blue is the formal experimental sample.

3.2 Presentation of QC samples in PCA-X one-dimensional distribution map

we can see that the QC samples are all within ±2STD in Figure S8, indicating that the quality of the experimental data is very high.

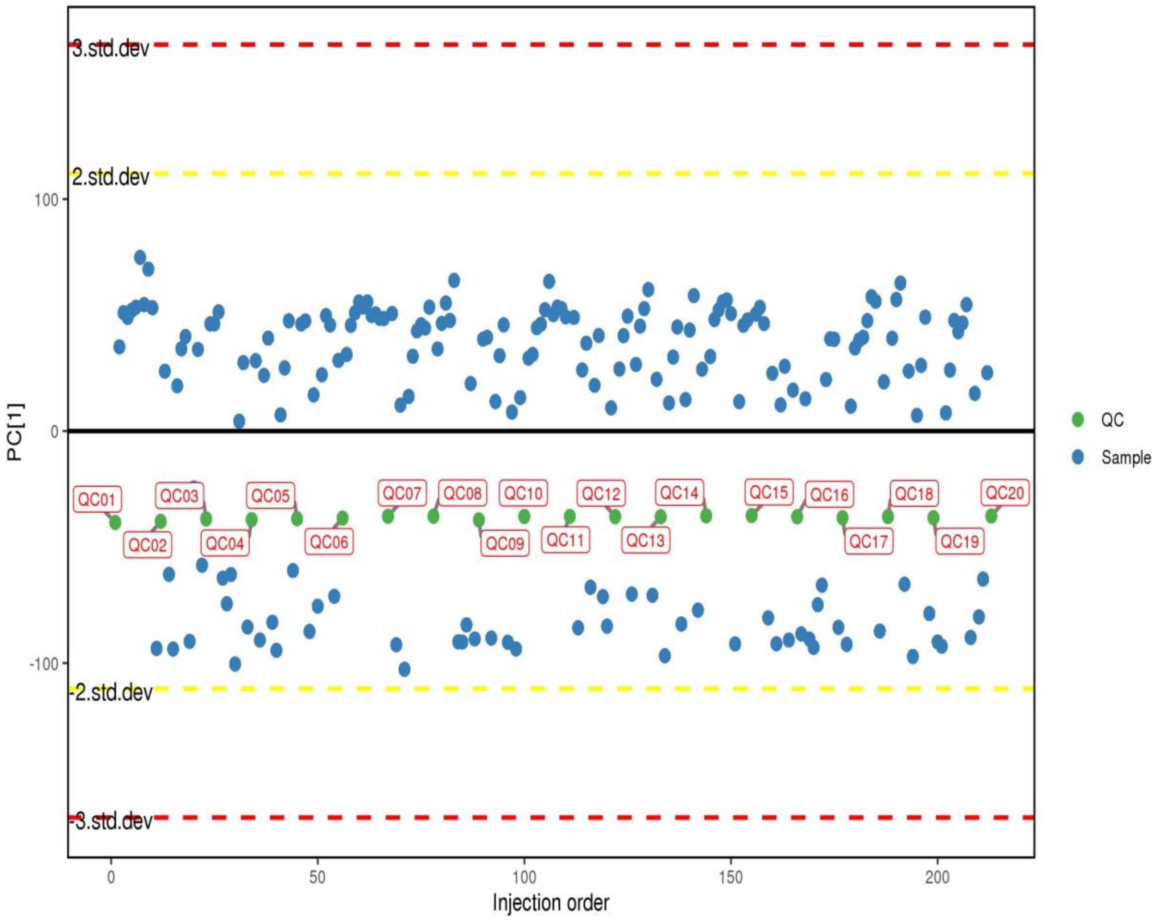

Figure S8. One-dimensional PCA-X distribution map of QC samples

3.3 Correlation of QC samples

Figure S9 shows that the correlation of QC samples is very high, indicating that the data quality of this experiment is very good.

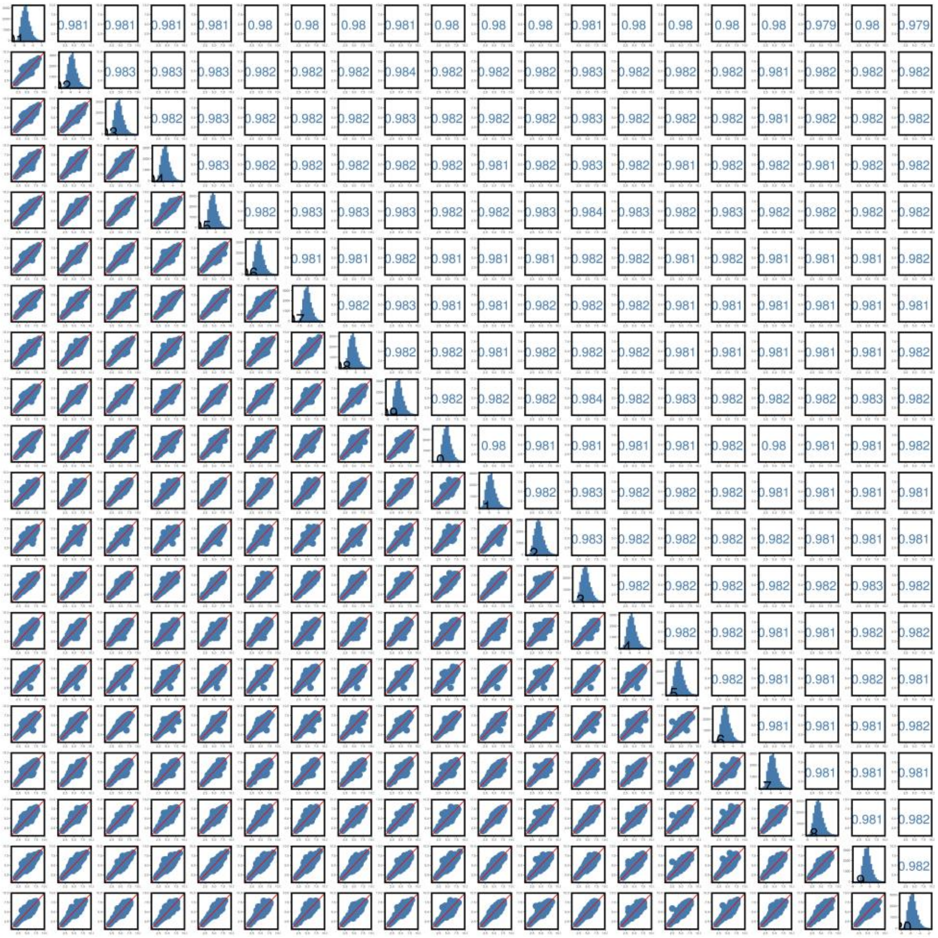

Figure S9. Correlation analysis of QC samples

3.4 Stability of internal standard response in QC samples

The internal standard is the introduced isotope labeled metabolite, and the internal standard concentration of the QC sample is the same, so the smaller the response difference of the internal standard is (median RSD ≤ 10%), indicating that the more stable the system is, the higher the data quality is. From the data in the Table, we can see that the experimental data are of high quality.

Stability of internal standard response in QC samples

nan	rt	mz	rsd
**IS1**	224.9	121.0445	0.0409
**IS2**	119.5	183.0822	0.0109
**IS3**	163.1	133.1061	0.0126
**IS4**	219.7	110.108	0.0288
**IS5**	197.1	85.1318	0.0064
**IS6**	29.3	127.0798	0.0296
