# Supplementary figures and images for: Nontargeted and targeted metabolic profile of metabolic syndrome patients: a study based on Yi and Han populations in Yunnan
Source: Front Endocrinol (Lausanne). 2025 May 14;16:1488099. doi: 10.3389/fendo.2025.1488099 (PMC12116332; doi:10.3389/fendo.2025.1488099)

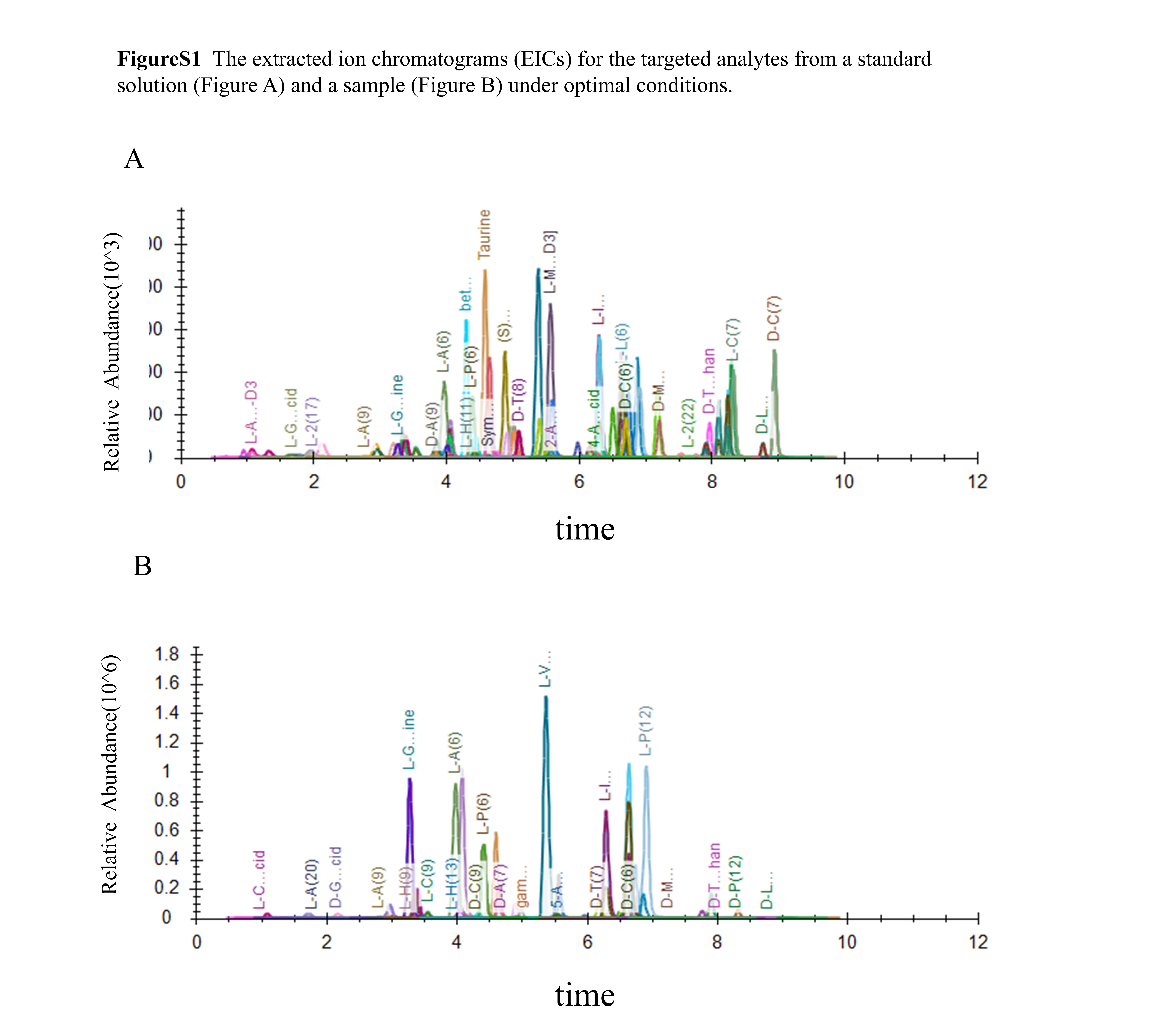

Supplement: Supplementary file 1 [file DataSheet1.zip › 1488099_SupMaterial/Figure S1-EICs for the targeted analytes.tif]
